# Sympathetic Activation Promotes Kidney Fibrosis in Mice via Macrophage‐Derived N2ICD‐Enriched Extracellular Vesicles

**DOI:** 10.1002/advs.202504607

**Published:** 2025-09-04

**Authors:** Huiwen Ren, Yayan Hou, Chengsen Mu, Yinghong Zheng, Yuanyang Wang, Shumin Guo, Lu Wang, Wenlong Shang, Zhuming Yin, Cheng Dong, Yongsheng Chang, Bin Zhou, Renjie Chai, Ying Yu, Yujun Shen

**Affiliations:** ^1^ Department of Pharmacology, Tianjin Key Laboratory of Inflammatory Biology, Center for Cardiovascular Diseases, Haihe Laboratory of Cell Ecosystem, Key Laboratory of Immune Microenvironment and Disease (Ministry of Education), The Province and Ministry Co‐sponsored Collaborative Innovation Center for Medical Epigenetics, Key Laboratory of Experimental Hematology, School of Basic Medical Sciences Tianjin Medical University Tianjin 300070 China; ^2^ Department of Bioinformatics, Tianjin Key Laboratory of Inflammation Biology, School of Basic Medical Sciences Tianjin Medical University Tianjin 300070 China; ^3^ Department of Breast Oncoplastic Surgery, Tianjin Medical University Cancer Institute and Hospital; National Clinical Research Center for Cancer; Key Laboratory of Breast Cancer Prevention and Therapy, Tianjin Medical University, Ministry of Education; Key Laboratory of Cancer Prevention and Therapy, Tianjin; Tianjin's Clinical Research Center for Cancer Sino‐Russian Joint Research Center for Oncoplastic Breast Surgery Tianjin 300060 China; ^4^ Department of Biochemistry and Molecular Biology, The Province and Ministry Co‐sponsored Collaborative Innovation Center for Medical Epigenetics, Key Laboratory of Immune Microenvironment and Disease (Ministry of Education), School of Basic Medical Sciences Tianjin Medical University Tianjin 300070 China; ^5^ Department of Physiology and Pathophysiology, Key Laboratory of Immune Microenvironment and Disease (Ministry of Education), Tianjin Key Laboratory of Cellular Homeostasis and Disease, School of Basic Medical Sciences Tianjin Medical University Tianjin 300070 China; ^6^ CAS CEMCS‐CUHK Joint Laboratory, New Cornerstone Science Laboratory, Key Laboratory of Multi‐Cell Systems, Shanghai Institute of Biochemistry and Cell Biology, Center for Excellence in Molecular Cell Science, Chinese Academy of Sciences University of Chinese Academy of Sciences Shanghai 200031 China; ^7^ Key Laboratory for Developmental Genes and Human Disease, Ministry of Education, Institute of Life Sciences Southeast University Nanjing 210096 China

**Keywords:** α2B‐adrenoceptor, extracellular vesicles, macrophages, notch2, renal fibrosis, sympathetic nerve activity

## Abstract

Persistent overactivation of the renal sympathetic nervous system drives kidney inflammation and fibrosis. Macrophages contribute to fibrogenesis by secreting various pro‐fibrogenic mediators. However, whether the sympathetic nervous system regulates renal fibrosis by modulating macrophage‐fibroblast interaction remains unclear. Here, it is demonstrated that norepinephrine (NE)‐treated macrophages promoted renal fibroblast activation through the transfer of Notch2 intracellular domain (N2ICD)‐enriched extracellular vesicles (EVs) to fibroblasts. Depletion of macrophage mitigated kidney fibrosis in mice subjected to unilateral nephrectomy plus contralateral ischemia‐reperfusion injury (Npx‐IRI) or repeated low‐dose cisplatin (RLDC) regimen. Macrophage‐specific deletion of Notch2 or α2B‐adrenoceptor disrupted N2ICD‐EV formation and protected mice from kidney fibrosis. Mechanistically, N2ICD stabilized Smad3 by preventing its ubiquitin‐dependent degradation, thereby enhancing TGF‐β signaling to promote fibroblast activation. These findings establish a sympathetic nerve‐macrophage‐fibroblast axis in renal fibrosis and highlight macrophage‐specific Notch2 inhibition as a potential therapeutic strategy.

## Graphical abstract

Norepinephrine/α2B‐adrenoceptor axis promotes the secretion of Notch2 intracellular domain (N2ICD)‐enriched extracellular vesicles (EVs) from macrophages. These EVs deliver N2ICD into fibroblasts, thereby inducing their activation. Deletion of Notch2 or α2B‐AR in macrophages disrupts N2ICD‐enriched EV formation and alleviates kidney fibrosis in mice. Mechanistically, N2ICD stabilizes Smad3 by preventing its ubiquitin‐mediated degradation, enhancing TGF‐β signaling, and promoting fibroblast activation.

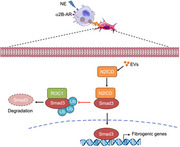

## 1 Introduction

Kidney fibrosis is a common pathological feature of chronic kidney disease (CKDs), and a major determinant of progressive renal failure. Renal fibrotic lesions arise from an injured microenvironment, called the fibrogenic niche, primarily composed of kidney residents such as the renal nerve, renal parenchymal cells, fibroblasts, and infiltrated inflammatory cells. The activation of renal fibroblasts in the niche is a key step in the development of fibrosis in the kidney.^[^ ^1^ ^]^ Pro‐fibrotic factors from the injured tubular epithelia, as well as inflammatory cells, can trigger the activation of renal fibroblasts in a paracrine or autocrine manner.^[^ ^2^ ^]^ However, the cellular interactions and molecular pathways governing these processes remain incompletely understood.

Renal sympathetic efferent and afferent nerves, which lie adjacent to the intrarenal vasculature, are crucial for the regulation of the fibrogenic microenvironment. In both patients and experimental animals with CKD, sympathetic nerve activity is markedly increased, accompanied by elevated levels of neurotransmitters such as norepinephrine (NE) in renal tissues.^[^ ^3^, ^4^, ^5^, ^6^, ^7^ ^]^ Studies have shown that renal denervation significantly alleviates renal fibrosis in mouse kidneys following unilateral ischemia‐reperfusion injury.^[^ ^8^ ^]^ Remarkably, local infusion of NE into denervated kidneys can recapitulate the pro‐fibrogenic phenotype observed in innervated kidneys.^[^ ^7^ ^]^ Epinephrine and NE exert their effects by binding to adrenoceptors (ARs), which are expressed on nephron segments as well as on immune cells, including T cells, macrophages, and B cells.^[^ ^9^, ^10^ ^]^ The renal sympathetic system has been shown to exacerbate CKD progression by modulating the inflammatory reflex.^[^ ^10^ ^]^ For instance, increased sympathetic outflow activates T cells via NE, thereby promoting renal inflammation and hypertension.^[^ ^11^ ^]^ However, the precise neural‐immune mechanisms driving kidney fibrosis require further elucidation.

Inflammation plays a central roles in fibrogenesis.^[^ ^2^ ^]^ Infiltration of inflammatory cell is a defining feature of the fibrotic niche and is closely associated with interstitial fibrosis and tubular atrophy.^[^ ^12^ ^]^ Among these inflammarory cells, macrophages play a pivotal role in the initiation and progression of renal fibrosis, as evidenced by the marked reduction in collagen deposition observed in obstructed murine kidneys following macrophage depletion.^[^ ^12^, ^13^ ^]^ In particular, they have been shown to provide direct signals to fibroblasts, promoting their differentiation into myofibroblast.^[^ ^14^ ^]^ Recent evidence highlights extracellular vesicles (EVs), membrane‐bound vesicle secreted by macrophages, as critical mediators in the fibrotic process across various tissues, including the lung,^[^ ^15^, ^16^ ^]^ liver,^[^ ^17^, ^18^ ^]^ and heart.^[^ ^19^ ^]^ These EVs carry bioactive molecules, including proteins, lipids, mRNA, DNA, and small non‐coding RNAs, with their cargo composition largely dependent on the nature of the stimulus and activation stage of macrophages, thereby enabling intercellular communication.^[^ ^20^ ^]^ For instance, EVs‐derived from macrophages under high glucose condition have been shown to deliver TGF‐β1 mRNA to mesangial cells, thereby promoting excessive extracellular matrix (ECM) production and cellular proliferation via activation of TGF‐β1 signaling.^[^ ^21^ ^]^ Despite these advances, the specific component of macrophage‐derived EVs and their precise roles in kidney fibrosis remain incompletely characterized and warrant further investigation.

In this study, we hypothesize that overactive renal sympathetic nerves promote the release of pro‐fibrotic EVs from macrophages, driving kidney fibrosis. To test this hypothesis, we employed a proteomic approach to profile the components of macrophage‐derived EVs following NE treatment, identified the key EV component that promotes renal fibroblast activation in mouse kidney fibrosis models, and further investigated the underlying mechanisms by which this component facilitates fibroblast activation.

## 2 Results

### 2.1 NE‐Stimulated Macrophages Secrete EVs to Induce Renal Fibroblast Activation

**Figure 1:**
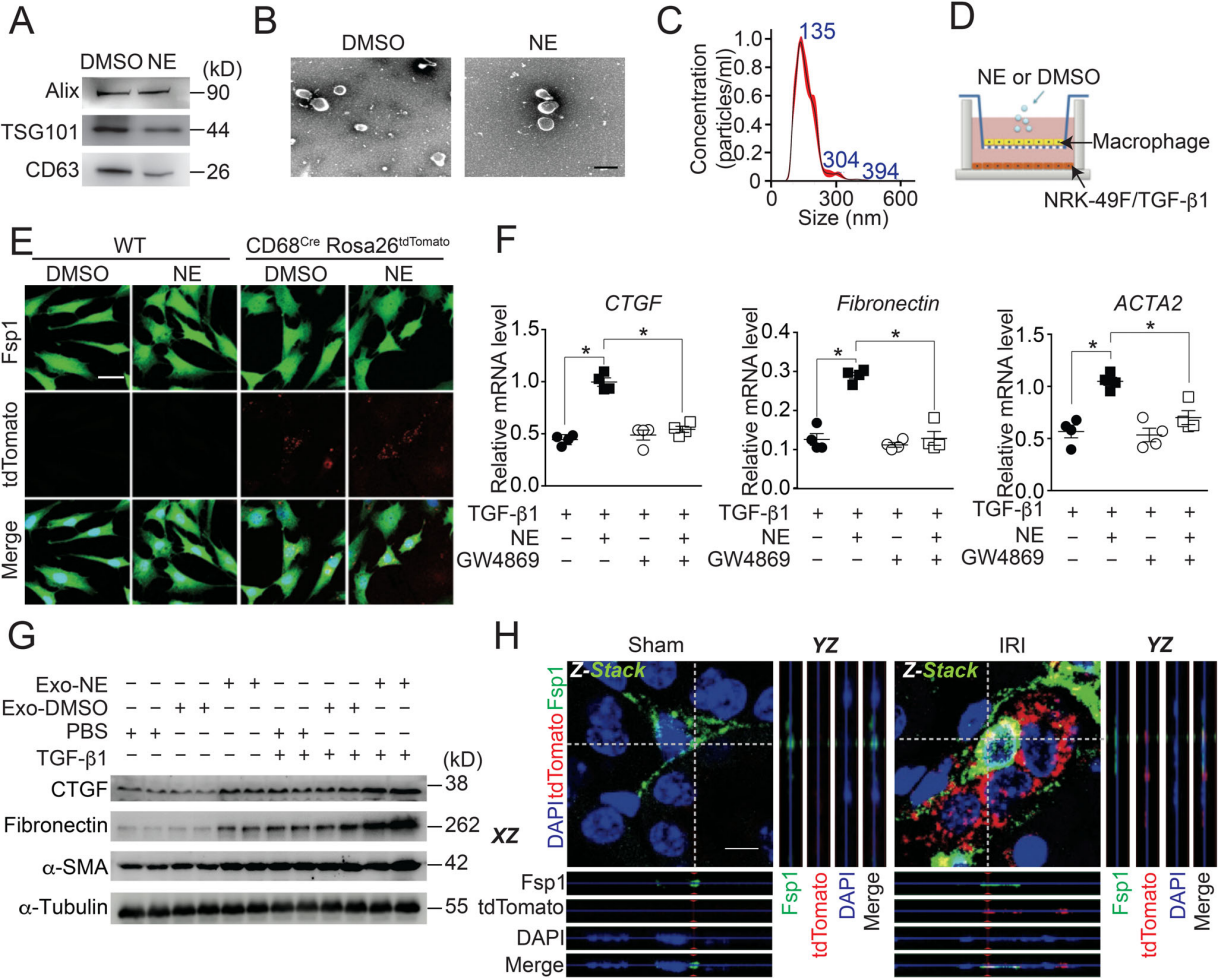
EVs from NE‐treated macrophages promotes renal fibroblast activation. A) Immunoblot analysis of markers (Alix, TSG101 and CD63) in EVs from DMSO‐ or NE‐treated primary macrophages isolated from the mouse peritoneal cavity. B) Representative micrographs from transmission electron microscopy of purified EVs from DMSO‐ or NE‐treated primary macrophages. Scale bar: 100 nm. C) Particle size distribution of EVs from NE‐treated primary macrophages. D) Co‐culture system for investigation of the interactions between primary macrophages and renal fibroblasts (NRK‐49F). E) Representative images from confocal fluorescence microscopy of macrophage‐derived EVs (red) uptake by NRK‐49F cells (green). Scale bar: 10 µm. F) Relative fibrogenic gene expression (CTGF, Fibronectin and ACTA2) in TGF‐β1‐treated NRK‐49F cells after co‐cultured with NE‐exposed primary macrophages with or without GW4869 treatment. n = 4. G) Immunoblot analysis of CTGF, Fibronectin and α‐SMA expression in TGF‐β1‐treated NRK‐49F cells in the presence or absence of EVs isolated from NE‐exposed primary macrophages. H) Representative Z‐stack images from confocal fluorescence microscopy of the intracellular localization of macrophages‐derived EVs with tdTomato (red) in renal fibroblasts (green) of Npx‐IRI CD68^Cre^ Rosa26^tdtomtato^ mice. *XZ* and *YZ* indicate signals from dotted lines on Z‐stack images. Scale bar: 5 µm. Data represent mean ± standard error of mean (SEM). Statistical significance was evaluated by Two‐way ANOVA, followed by Tukey's test for multiple comparisons (F). * *p* < 0.05.

Macrophage‐derived EVs have been previously implicated in intercellular communication and multiple biological functions.^[^ ^22^ ^]^ We then isolated EVs from NE‐treated macrophages, confirming EV markers (Alix, TSG101, and CD63), typical ultrastructure, and size distribution (**Figure** **1**A–C). To assess their role in renal fibrosis, we co‐cultured NE‐treated macrophage with renal fibroblast cells(normal rat kidney‐49F, NRK‐49F) to assess whether macrophage‐derived EVs could directly induce fibroblast activation (Figure 1D). Compared with fibroblasts co‐cultured with wild‐type (WT) macrophages, those co‐cultured with macrophages isolated from CD68^Cre^Rosa26^tdtomtato^ mice exhibited clear tdTomato signals in their cytoplasm, regardless of NE treatment on macrophages. This observation indicates that macrophage‐derived EVs can be effectively taken up by fibroblasts. (Figure 1E). Notably, NE did not significantly alter the quantity of EVs secreted by macrophages (Figure S1, Supporting Information); however, conditioned medium from NE‐treated macrophages markedly upregulated the expression of fibrogenic genes, including connecitve tissue growth factor(CTGF), Fibronectin, and actin alpha2 (ACTA2) in NRK‐49F cells (Figure 1F). GW4869, an potent inhibitor of exosome biogenesis and formation, markedly reduced EV secretion (Figure S1, Supporting Information) and abolished NE‐induced fibroblast activation (Figure 1F). Moreover, incubation of NRK‐49F cells with EVs isolated from NE‐treated macrophages markedly promoted the expression of fibrogenesis proteins such as CTGF, Fibronectin, and α‐smooth muscule actin (α‐SMA) and enhanced the pro‐fibrotic effect of TGF‐β1 in NRK‐49F cells (Figure 1G). In a mouse model of unilateral nephrectomy plus contralateral ischemia‐reperfusion injury (Npx‐IRI), we also observed that renal fibroblasts (green, Fsp1‐positive) are surrounded by tdTomato‐labeled macrophages (red). Notably, subcellular co‐localization of tdTomato signals within Fsp1‐positive fibroblasts (Figure 1H) suggests that macrophage‐derived tdTomato‐labeled extracellular vesicles were successfully translocated into renal fibroblasts. Thus, macrophage‐derived EVs promote the activation of renal fibroblasts upon NE stimulation.

### 2.2 N2ICD is Identified as a Pro‐Fibrotic Component in EVs from NE‐Treated Macrophages

**Figure 2:**
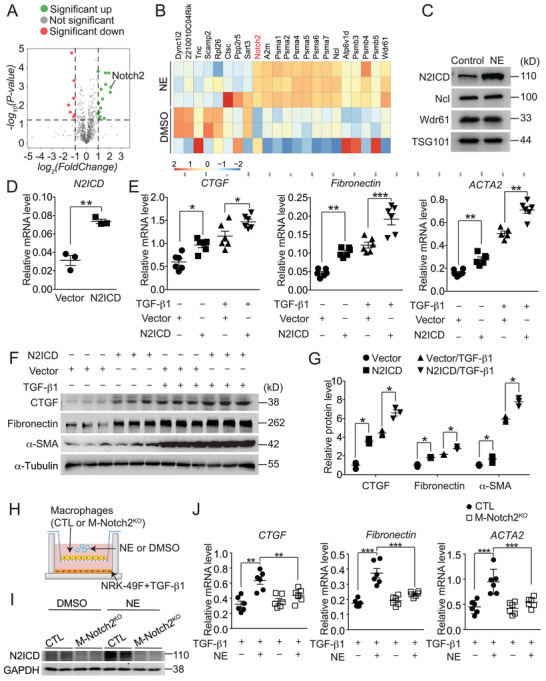
N2ICD acts as a pro‐fibrotic component of EVs secreted by NE‐treated macrophages. A) Volcano plot of differential expressed proteins in EVs from primary macrophages with NE or DMSO treatment. B) Heatmap presenting differential expressed proteins in EVs from primary macrophages with NE or DMSO treatment. C) Immunoblot analysis of N2ICD, Ncl and Wdr61 in EVs from primary macrophages with or without NE treatment. D) RT‐PCR analysis of the expression of N2ICD in NRK‐49F cells infected with N2ICD‐packaged lentivirus. n = 3. E) Effect of N2ICD‐overexpression on fibrogenic gene expression (CTGF, Fibronectin and ACTA2) in TGF‐β1‐treated NRK‐49F cells. n = 5–6. F) Immunoblot analysis of CTGF, Fibronectin and α‐SMA expression in TGF‐β1‐treated NRK‐49F cells with or without N2ICD overexpression. G) Quantification of relative protein levels in F. n = 3. H) Co‐culture system for investigation of the interaction between macrophages and NRK‐49F cells. I) Immunoblot analysis of N2ICD expression in NRK‐49F cells co‐cultured with NE‐treated macrophage from CTL or M‐Notch2^KO^. J) Relative mRNA levels of CTGF (*left*), Fibronectin (*middle*) and ACTA2 (*right*) in TGF‐β1‐treated NRK‐49F cells when co‐cultured with Notch2 deficient macrophages with NE treatment. n = 6. Data represent mean ±SEM. Statistical significance was evaluated by Mann–Whitney U tests (D), One‐way ANOVA, followed by Tukey's test for multiple comparisons (E,G) and Two‐way ANOVA, followed by Tukey's test for multiple comparisons (J). * *p* < 0.05; ** *p* < 0.01;*** *p* < 0.001.

To identify the pro‐fibrotic component in EVs from NE‐treated macrophages, we performed proteomic analysis on EVs isolated from macrophages and identified 15 upregulated and 7 downregulated proteins in EVs derived from NE‐treated macrophages compared to those from control cells (fold change ≥ 2, *p* < 0.01) (**Figure** **2**A,B). Since fibroblast‐to‐myoblast transition is primarily regulated by a cascade of transcription factors, we cross‐referenced these differentially expressed proteins with the Animal TFDB database and obtained three transcription‐related factors: Notch2 intracellular domain (N2ICD), Wdr61, and Ncl. Western blotting confirmed that N2ICD was upregulated in EVs from NE‐treated macrophages (Figure 2C). Notably, lentiviral overexpression of N2ICD (Figure 2D) markedly promoted the myofibroblast transition of NRK‐49F cells and amplified the pro‐fibrotic effects of TGFβ1(Figure 2E–G). To determine the functional role of macrophage‐derived N2ICD, we generated macrophage‐specific Notch2 knockout mice (M‐Notch2^KO^) (Supplemental Figure S2A,B). In co‐cultures of M‐Notch2^KO^ macrophages and NRK‐49F fibroblasts, Notch2 ablation abolished NE‐induced N2ICD accumulation in fibroblasts (Figure 2H,I) and significantly suppressed fibroblast activation (Figure 2J). These results suggest that NE‐stimulated macrophages drive renal fibroblast activation through transfer of N2ICD‐enriched EVs to fibroblasts.

### 2.3 Depletion of Macrophages Attenuates Renal Fibrosis in Mice

**Figure 3:**
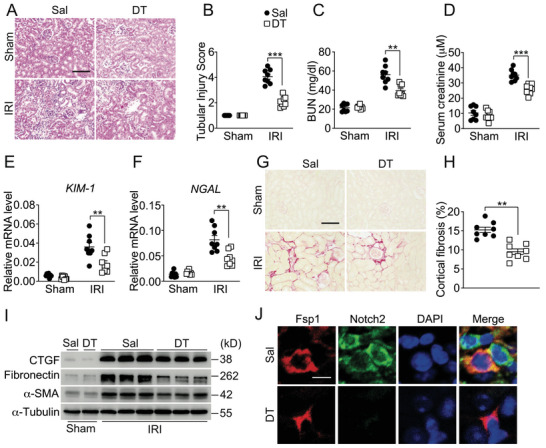
Depletion of macrophages protects against Npx‐IRI‐induced renal fibrosis in mice. A,B) Hematoxylin & Eosin (H&E) staining (A) and tubular injury score (B) of kidneys from LysM^Cre^/Rosa26^iDTR^Rosa26^iGFP^ mice underwent Npx‐IRI after DT administration. Scale bar: 100 µm. n = 8. C,D) Blood urea nitrogen (C) and creatinine (D) levels of LysM^Cre^/Rosa26^iDTR^Rosa26^iGFP^ mice underwent Npx‐IRI after DT administration. n = 8. E,F) Relative mRNA levels of KIM‐1 (E) and NGAL (F) in kidneys from LysM^Cre^/Rosa26^iDTR^Rosa26^iGFP^ mice underwent Npx‐IRI after DT administration. n = 8. G,H) Sirius red staining (G) of kidneys from LysM^Cre^/Rosa26^iDTR^Rosa26^iGFP^ mice underwent Npx‐IRI after DT administration and its quantification (H). Scale bar: 100 µm. n = 8. I) Immunoblot analysis of CTGF, Fibronectin and α‐SMA expression in kidneys from LysM^Cre^/Rosa26^iDTR^Rosa26^iGFP^ mice underwent Npx‐IRI after DT administration. J) Representative images from confocal fluorescence microscopy of the co‐localization of Notch2 and renal fibroblasts (Fsp1^+^) in kidneys from LysM^Cre^/Rosa26^iDTR^Rosa26^iGFP^ mice underwent Npx‐IRI after DT administration. Scale bar: 20 µm. Data represent mean ±SEM. Statistical significance was evaluated by Mann–Whitney U tests (H) and Two‐way ANOVA, followed by Tukey's test for multiple comparisons (B–F). ** *p* < 0.01;*** *p* < 0.001.

Sympathetic nerve activity is increased upon experimental kidney injury, such as ischemia‐reperfusion injury (IRI).^[^ ^7^ ^]^ Consistent with previous study, unilateral nephrectomy plus contralateral ischemia‐reperfusion injury (Npx‐IRI) significantly upregulated renal NE levels (Figure S3, Supporting Information). Critically, renal denervation markedly reduced kidney injury and fibrosis in mice after Npx‐IRI insults (Figure S4, Supporting Information). To explore the precise role of macrophage‐derived Notch2 in the progression of sympathetic nerve activity‐associated renal fibrosis, we expressed the diphtheria toxin (DT) receptor in transgenic mice under control of the LysM promoter (LysM^Cre^/Rosa26^iDTR^Rosa26^iGFP^). Administration of DT caused an ≈90% reduction in the renal accumulation of macrophages (Figure S5A,B, Supporting Information). Macrophage depletion significantly attenuated Npx‐IRI‐induced renal dysfunction in mice, as determined by improved histological damage score (**Figure** **3**A,B), decreased serum urea and creatinine levels (Figure 3C,D), as well as the renal injury gene expression such as KIM‐1 and NGAL (Figure 3E,F). Accordingly, macrophage depletion markedly reduced the renal fibrotic area (Figure 3G,H) and the protein expression of pro‐fibrotic markers, including α‐SMA, CTGF, and Fibronectin in the injured kidney (Figure 3I). More importantly, we observed almost null Notch2 transfer to the renal fibroblasts in Npx‐IRI kidneys of macrophage‐depleted mice (Figure 3J). Additionally, consistent with our Npx‐IRI results, repeated low‐dose cisplatin (RLDC) treatment also induced a significant increase of NE levels in mouse kidneys (Figure S6, Supporting Information) and macrophage depletion protected kidneys from functional and histological deterioration (Figure S7A–F, Supporting Information) and retarded the development of fibrosis in LysM^Cre^/Rosa26^iDTR^Rosa26^iGFP^ mice after RLDC treatment (Figure S7G–I, Supporting Information). Thus, macrophages play a profibrotic role in kidney fibrosis, and macrophage‐derived Notch2 may act as a major contributor to the renal fibrosis.

### 2.4 Macrophage‐Specific Deletion of Notch2 Protects Against Npx‐IRI or RLDC‐Induced Kidney Fibrosis in Mice

**Figure 4:**
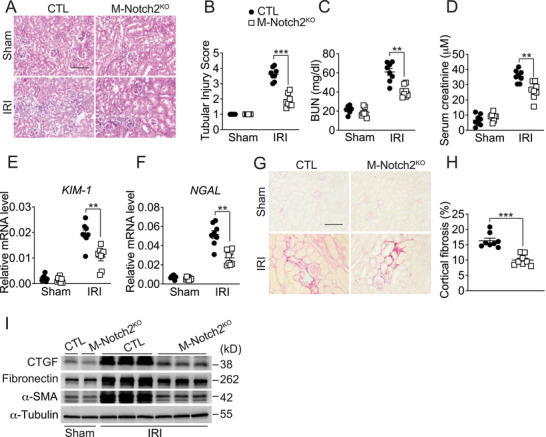
Ablation of Notch2 in macrophages attenuates Npx‐IRI‐induced renal fibrosis in mice. A,B) H&E staining (A) and tubular injury score (B) of kidneys from Npx‐IRI M‐Notch2^KO^ mice. Scale bar: 100 µm. n = 8. C,D) Blood urea nitrogen (C) and creatinine (D) levels of Npx‐IRI Notch2^KO^ mice. n = 8. E,F) Relative mRNA levels of KIM‐1 (E) and NGAL (F) in kidneys from Npx‐IRI Notch2^KO^ mice. n = 8. G,H) Representative images (G) and quantification (H) of Sirius red staining of kidneys from Npx‐IRI Notch2^KO^ mice. Scale bar: 100 µm. n = 8. I) Immunoblot analysis of CTGF, Fibronectin, and α‐SMA expression in kidneys from Npx‐IRI M‐Notch2^KO^ mice. Data represent mean ±SEM. Statistical significance was evaluated by Mann–Whitney U tests (H) and Two‐way ANOVA, followed by Tukey's test for multiple comparisons (B–F). ** *p* < 0.01; *** *p* < 0.001.

In a mouse model of Npx‐IRI, macrophage‐specific Notch2 deletion markedly alleviated renal histopathological injury (**Figure** **4**A,B), reduced serum urea and creatinine levels (Figure 4C,D), and diminished the expression of renal injury marker genes (Figure 4E,F). Consistently, Npx‐IRI‐induced tubulointerstitial fibrosis (Figure 4G,H) and fibrotic protein expression in the kidney (Figure 4I) were markedly decreased in M‐Notch2^KO^ mice compared to control mice. Moreover, Notch2 deficiency in macrophages also protected mice RLDC‐induced renal injury (Figure S8A–F, Supporting Information) and attenuated the subsequent kidney fibrosis in mice (Figure S8G–I, Supporting Information). Therefore, macrophage‐derived Notch2 is indispensable for the development of injury‐induced kidney fibrosis.

Next, we investigated whether fibroblast‐derived Notch2 also contributed to the pathogenesis of kidney fibrosis. Single‐cell RNA sequencing revealed that Notch2 was predominantly expressed in macrophages whereas was barely detected in fibroblasts from both mouse (GSE:139 107) and human kidneys (GSE:134 355) (Figure S9A,B, Supporting Information). Strikingly, renal IRI insult had no obvious impact on Notch2 gene expression in mouse kidney fibroblasts (GSE:139 107) (Figure S9C, Supporting Information), and depletion of macrophages completely eliminated Notch2 protein expression in renal fibroblasts from Npx‐IRI mice (Figure S9D, Supporting Information). Consistently, the loss of Notch2 in fibroblasts (Figure S10, Supporting Information) had no obvious effects on Npx‐IRI‐induced renal structural and functional damage (Figure S11A–F, Supporting Information), as well as renal collagen deposition in mice (Figure S11G–I, Supporting Information). However, ablation of Notch2 in both macrophages and fibroblasts conferred comparable protective effect against Npx‐IRI‐induced kidney fibrosis in mice (Figure S11, Supporting Information). On the contrary, ectopic expression of N2ICD in fibroblasts (Figure S12, Supporting Information) caused spontaneous renal interstitial collagen deposition and exaggerated Npx‐IRI‐induced renal fibrosis in mice (Figure S13, Supporting Information). Taken together, our data indicate that Notch2 derived from macrophages promotes kidney fibrogenesis by activating renal fibroblasts.

### 2.5 Macrophage α2B‐AR/Notch2 Axis Governs Fibrogenesis in Mouse Kidneys

**Figure 5:**
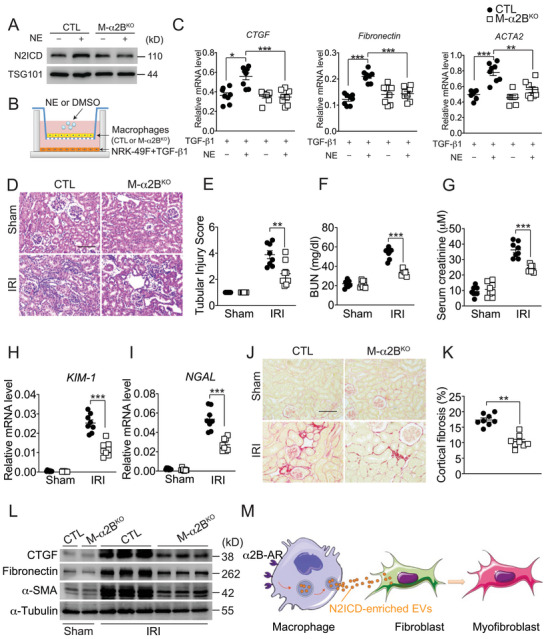
Macrophage‐specific α2B‐AR deletion alleviates Npx‐IRI‐induced renal fibrosis in mice. A) Immunoblot analysis of N2ICD levels in EVs from NE‐treated macrophage isolated from M‐α2B^KO^ mice. B) Co‐culture system for investigation of the effect of EVs from α2B deficient macrophages on the activation of NRK‐49F cells. C) Relative mRNA levels of CTGF (*left*), Fibronectin (*middle*), and ACTA2 (*right*) in TGFβ1‐treated NRK‐49F cells when co‐cultured with NE‐exposed α2B‐deficient macrophages. n = 8. D,E) H&E staining (D) and tubular injury score (E) of kidneys from Npx‐IRI M‐α2B^KO^ mice. Scale bar: 100 µm. n = 8. F,G) Blood urea nitrogen (F) and creatinine (G) levels of Npx‐IRI M‐α2B^KO^ mice. n = 8. H,I) Relative mRNA levels of KIM‐1 (H) and NGAL (I) in kidneys from Npx‐IRI M‐α2B^KO^ mice. n = 8. J,K) Sirius red staining (J) of kidneys from Npx‐IRI M‐α2B^KO^ mice and its quantification (K). Scale bar: 100 µm. n = 8. L) Immunoblot analysis of CTGF, Fibronectin and α‐SMA expression in kidneys from Npx‐IRI M‐α2B^KO^ mice. M) Schematic model of sympathetic nervous activation regulates renal fibrosisby modulating macrophage‐fibroblast communications via N2ICD‐enriched EVs derived from macrophages. Data represent mean ±SEM. Statistical significance was evaluated by Mann–Whitney U tests (K) and Two‐way ANOVA, followed by Tukey's test for multiple comparisons (C, E, F, G, H, I). * *p* < 0.05;** *p* < 0.01; *** *p* < 0.001.

Since sympathetic adrenergic nerves modulates renal function via adrenoceptors, we then examined which adrenoceptor mediated the N2ICD‐containing EV release from macrophages in response to NE. Adrenergic receptors are classified into two families, α and β, which further are divided into α1 (α1A, α1B, and α1D), α2 (α2A, α2B, and α2C) family, and three β subtypes (β1, β2, and β3).^[^ ^23^ ^]^ We found that α1A, α2B, and β2 subtypes were abundantly expressed in macrophages (Figure S14A, Supporting Information). Silencing of α2B, but not α1A and β2, in macrophages (Figure S14B, Supporting Information) completely abolished NE‐induced renal fibroblast activation in the macrophage/fibroblast co‐culture system (Figure S14C,D, Supporting Information). Next, we generated macrophage‐specific α2B‐AR deficient mice (M‐α2B^KO^) to explore the role of macrophage α2B‐AR in renal fibrosis (Figure S15A–C, Supporting Information). Deletion of α2B‐AR in macrophages prevented the NE‐induced accumulation of N2ICD in macrophage‐derived EVs (**Figure** **5**A), and abolished NE‐treated EV‐ mediated activation of renal fibroblast (Figure 5B,C). In line with the in vitro results, α2B‐AR deficiency in macrophages significantly improved Npx‐IRI‐induced renal dysfunction and tissue structural damage (Figure 5D–I) and reduced subsequently kidney fibrosis in mice (Figure 5J–L). Again, ablation of α2B‐AR in macrophages markedly attenuated RLDC‐induced renal injury and inhibited renal fibrosis in mice (Figure S16, Supporting Information). Collectively, these results support a model in which the NE/α2B‐AR axis promotes the secretion of N2ICD‐enriched EVs from macrophages, which are subsequently transferred to renal fibroblasts to drive their activation and contribute to the development of renal fibrosis (Figure 5M).

### 2.6 N2ICD Stabilizes Smad3 Protein and Enhances its Transcriptional Activity in Renal Fibroblasts

**Figure 6:**
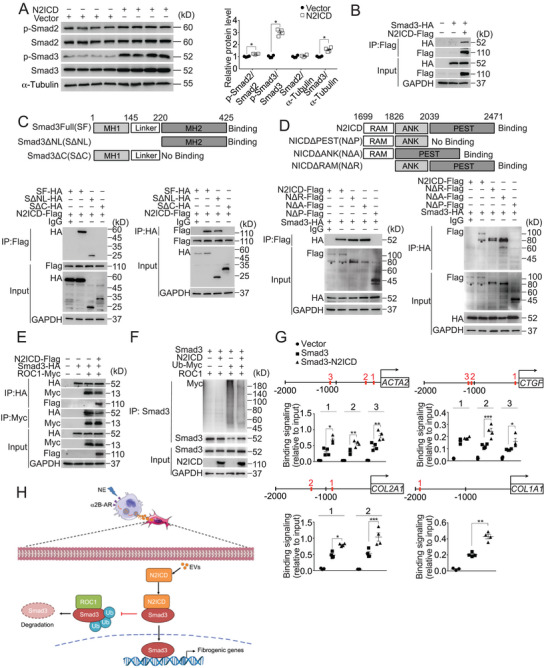
N2ICD activates renal fibroblasts by stabilizing Smad3 via binding to its MH2 domain. A) Immunoblot analysis (*left*) and quantification (*right*) of p‐Smad2, Smad2, p‐Smad3, and Smad3 protein expression in NRK‐49F cells infected with N2ICD‐packaged lentivirus. n = 4. B) Co‐IP analysis of physical interactions between N2ICD and Smad3 in NIH 3T3 cells. C) Strategy for Smad3 truncations (*top*) and Co‐IP analysis of the binding domain of Smad3 with N2ICD (*bottom*). *bottom left*: IP with anti‐Flag; *bottom right*: IP with anti‐HA. SF: full length; S△NL: MH2 domain; S△C: MH1 domain and linker. D) Strategy for Flag‐N2ICD truncation (*top*) and Co‐IP analysis of the binding domain of N2ICD with Smad3 (*bottom*). *bottom left*: IP with anti‐Flag; *bottom right*: IP with anti‐HA. N2ICD: full length; N△P:RAM and ANK domains; N△A: RAM and PEST domains; N△R: ANK and PEST domains. * non‐specific bands. E) Co‐IP analysis of the effect of N2ICD on the interaction between ROC1 and Smad3 in NIH 3T3 cells. F) Effect of N2ICD on ubiquitin‐dependent degradation of Smad3 mediated by ROC1 in NIH 3T3 cells. G) ChIP analysis of the effect of N2ICD on the binding of Smad3 within the promoter region of fibrogenic genes in NRK‐49F cells, including ACTA2 (*top left*), CTGF (*top right*), COL2A1 (*bottom left*), and COL1A1 (*bottom right*). n = 3–4. The red number (1/2/3) indicated the binding sit of Smad3 in the promoter region of fibrogenic genes. H) Schematic illustration of N2ICD‐enriched EVs derived from macrophages facilitating fibrogenic gene transcription by stabilizing the Smad3 protein. Data represent the mean ±SEM. Statistical significance was evaluated using Mann–Whitney U tests (A) and One‐way ANOVA, followed by Tukey's test for multiple comparisons (G). * *p* < 0.05; ** *p* < 0.01; *** *p* < 0.001.

TGF‐β1‐Smad2/3 signaling pathway is a major inducer of renal fibroblast activation.^[^ ^24^ ^]^ Interestingly, we found that N2ICD overexpression selectively increased the protein levels of phosphorylated and total Smad3 in renal fibroblasts (**Figure** **6**A) but had no obvious influence on its mRNA expression levels (Figure S17, Supporting Information). Moreover, the Co‐IP assay demonstrated a physical interaction between N2ICD and Smad3 (Figure 6B). Smad3 is composed of two conserved functional domains, the NH2‐terminal mad homology(MH)1 domain and the COOH‐terminal MH2 domain, separated by a non‐conserved linker.^[^ ^25^ ^]^ The MH2 domain can interact with regulator of cullins 1 (ROC1), a subunit of the SCF E3 ligase complex composed of Skp1, Cullins, and F‐box proteins, for Smad3 ubiquitination and degradation.^[^ ^26^ ^]^ We then constructed a series of truncated HA‐fused fragments, including Smad3 full‐length (SF), MH2 domain (S△NL), and MH1 domain with linker (S△C) (Figure 6C, top). Co‐IP assays revealed that the MH2 domain of Smad3, comprising amino acids 220–425, interacted with N2ICD (Figure 6C, bottom). We also constructed a series of truncated N2ICD fragments^[^ ^27^ ^]^ (Figure 6D, top) and observed that the proline‐glutamic acid‐serine‐threonine (PEST) domain of N2ICD directly bound to Smad3 (Figure 6D, bottom). More importantly, forced expression of N2ICD disrupted the Smad3‐ROC1 interaction, as evidenced by decreased binding of Smad3 to ROC1 (Figure 6E) and subsequently reduced ubiquitination‐mediated degradation of Smad3 (Figure 6F). Chromatin immunoprecipitation (ChIP) assay revealed that N2ICD dramatically enhanced Smad3 binding activity on the promoter region of fibrogenic genes, including ACTA2, CTGF, COL1A1, and COL2A1 in renal fibroblasts (Figure 6G). Conversely, Smad3 deletion abrogated N2ICD‐induced expression of Fibronectin, CTGF, and ACTA2, indicating that Smad3 is essential for the profibrotic activity of N2ICD (Figure S18, Supporting Information). Taken together, N2ICD promotes fibrogenesis in renal fibroblasts by competitively binding to the MH2 domain of Smad3 and preventing its ubiquitination and degradation, resulting in an enhanced TGF‐β signaling (Figure 6H).

## 3 Discussion

Overactivation of the renal sympathetic nervous system and the sustained inflammatory response play important roles in the progression of renal fibrosis. Here, we found that NE‐stimulated macrophages secreted N2ICD‐enriched EVs to activate renal fibroblasts. Deletion of adrenoceptor α2B‐AR or Notch2 in macrophages improved injury‐induced kidney fibrosis in mice, and N2ICD facilitated fibroblast activation by suppressing ubiquitination‐mediated degradation of Smad3. Thus, targeting α2B‐AR/Notch2 signaling could be an effective approach for the treatment of kidney fibrosis.

Renal sympathetic nerves play a crucial role in regulating renal physiological functions. In CKD, renal sympathetic activity is significantly amplified, accelerating the progression of kidney fibrosis.^[^ ^6^ ^]^ Blockade of sympathetic nerve‐derived signaling through renal denervation has been shown to substantially attenuate kidney fibrosis across various CKD models.^[^ ^7^, ^28^ ^]^ Notably, neurogenic NE signaling is implicated in driving renal inflammation and fibrogenesis.^[^ ^28^, ^29^ ^]^ Although the profibrotic effect of NE in hypertension and CKD have been partially attributed to tubular epithelial cell injury,^[^ ^9^ ^]^ our findings suggest that macrophage‐fibroblast communication mediated by N2ICD‐enriched EVs also plays a key role in sympathetic overstimulation‐induced kidney fibrosis. Similarly, macrophage‐derived EVs have been shown to facilitate fibroblast activation in pulmonary,^[^ ^30^ ^]^ liver,^[^ ^17^, ^31^ ^]^ and cardiac fibrosis.^[^ ^19^ ^]^ EVs have been extensively investigated in kidney fibrosis by transmitting pro‐fibrotic signals,^[^ ^32^, ^33^, ^34^ ^]^ and are emerging as potential diagnostic biomarkers and drug targets for renal fibrosis.^[^ ^35^ ^]^

Consistent with previous reports,^[^ ^13^ ^]^ we observed that depletion of macrophages significantly mitigated the fibrogenic response in the injured kidney. Indeed, both pro‐inflammatory (M1) and anti‐inflammatory (M2) macrophages contribute to renal fibrosis by secreting distinct sets of cytokines that influence fibroblast activation and extracellular matrix remodeling.^[^ ^12^, ^36^ ^]^ Macrophages express both α1/2‐ and β‐ARs,^[^ ^37^ ^]^ with activation of α2‐ARs known to promote a pro‐inflammatory macrophage phenotype.^[^ ^38^ ^]^ However, the specific role of individual α2‐AR subtypes remains largely unexplored. In our study, we found that among the α2‐AR subtypes, only α2B‐AR rapidly responded to macrophage‐mediated fibroblast activation. Deletion of α2B‐AR in macrophages significantly reduced fibrogenesis in mouse kidneys following Npx‐IRI, mirroring the anti‐fibrotic effects observed with α2‐AR antagonists in murine models of obstructive nephropathy.^[^ ^7^ ^]^ In addition, activation of α2B‐AR triggers Gβγ subunit‐dependent actin remodeling, a process essential for cargo sorting into EVs.^[^ ^39^ ^]^ Our identification of α2B‐AR^+^ macrophage as a major contributor in kidney fibrosis represents new insights into the mechanisms underlying sympathetic nerve‐mediated kidney fibrosis.

The Notch receptor family (Notch1‐4) belongs to highly conserved transmembrane proteins with diverse roles in modulating inflammation and fibrogenesis.^[^ ^40^, ^41^ ^]^ Although Notch expression is relatively low in mature human and rodent kidneys,^[^ ^42^ ^]^ its reactivation during CKD promotes kidney fibrosis.^[^ ^40^ ^]^ For instance, Notch1 expressed in injured podocytes drives renal fibrogenesis by inducing podocyte apoptosis.^[^ ^42^ ^]^ Notch3 is expressed in renal tubular epithelial epithelium in obstructive nephropathy,^[^ ^43^ ^]^ and its depletion protects against fibrosis.^[^ ^44^ ^]^ Similarly, Notch4 expression is elevated in the tubular epithelium of fibrotic kidneys in diabetic mice and humans, where it enhances fibrotic activity.^[^ ^45^ ^]^ In our study, Notch2 expression was enriched in renal macrophages, and ablation of Notch2 in macrophages protected mice from kidney fibrosis induced by Npx‐IRI‐ or RLDC. In agreement with our observations, inhibition of Notch signaling using N‐[N‐(3,5‐difluorophenacetyl)‐L‐alanyl]‐(S)‐phenylglycine t‐butyl ester^[^ ^46^, ^47^ ^]^ or myeloid‐specific disruption of the Notch transcriptional effector RBP‐J mitigates IRI‐induced renal injury and fibrosis.^[^ ^45^ ^]^ Genome‐wide expression analysis in human kidney samples have identified a strong correlation between Notch2 expression and the degree of interstitial fibrosis.^[^ ^48^ ^]^ Canonical Notch signaling involves cleavage of the Notch receptor to release the transcriptional regulator NICD, which can also be incorporated into EVs and transmitted as an intercellular message.^[^ ^49^ ^]^ We demonstrated that N2ICD is packaged into renal macrophage‐derived EVs and transferred to fibroblasts, and mediates pro‐fibrotic signaling via Smad3. Similarly, Notch regulates myofibroblast transition in synovial and pulmonary fibroblasts,^[^ ^50^, ^51^ ^]^ and acts as a downstream effector of TGF‐β signaling in maladaptive kidney regeneration.^[^ ^42^, ^52^ ^]^ Indeed, α2‐AR/Notch/Smad3 axis plays a critical role in regulating kidney function.^[^ ^53^, ^54^, ^55^ ^]^

In summary, we identified N2ICD‐enriched EV as a critical profibrotic component secreted by macrophages under sympathetic stimulation. Targeting macrophage N2ICD provides a novel therapeutic strategy to combat kidney fibrosis.

## 4 Experimental Section

Additional details for all methods are provided in the Supporting Information and Methods.

### Mice

Male C57BL/6 mice (6‐8‐week‐old) were used for all experiments, as male animals exhibited less variability in phenotype. All animal procedures were approved by the Laboratory Animal Management and Use Committee of the Tianjin Medical University. Details of the animal experiments can be found in the Supplementary Materials and Methods.

### Murine Models of Kidney Fibrosis

Detailed information on the preparation of the unilateral nephrectomy plus contralateral ischemia‐reperfusion injury (Npx‐IRI) model and the repeated low‐dose cisplatin (RLDC) model is provided in the Supporting Information.

### EV Isolation, Purification, and Analysis

Macrophage‐derived EVs were isolated and purified as previously described. Size distribution, morphology, and quantity were determined using electron microscopy and nanoparticle tracking analysis (NTA). Detailed methods are included in the Supplementary Materials and Methods.

### Statistical Analysis

All data are expressed as mean ± standard error of the mean (SEM). Data were analyzed using SPSS version 21.0 (IBM Inc., Armonk, NY, USA). Mann–Whitney U test was used to compare two independent samples. For comparisons of multiple groups, one‐way or two‐way ANOVA was used, followed by the *post hoc* Bonferroni test. Statistical significance was set at *p* < 0.05.

## Conflict of Interest

The authors declare no conflict of interest.

## Author Contributions

Y.Y. and Y.S. contributed equally to this work. H.R., Y.S., and Y.Y. designed the study; H.R., S.G., Y.H., W.S., C.M., and Y.W. performed the experiments; Y.Z., L.W., and Y.H. analyzed the data; H.R. wrote the paper; Z.Y., C.D., Y.C., B.Z., and R.C. provided experimental resource; Y.S. and Y.Y. critically revised the manuscript for key intellectual content.

## Supporting information



Supporting Information

Supporting Information

## Data Availability

The data that support the findings of this study are available from the corresponding author upon reasonable request.

## References

[1] L. Li , H. Fu , Y. Liu , Nat. Rev. Nephrol. 2022, 18, 545. 35788561 10.1038/s41581-022-00590-z

[2] W. Lv , G. W. Booz , Y. Wang , F. Fan , R. J. Roman , Eur. J. Pharmacol. 2018, 820, 65. 29229532 10.1016/j.ejphar.2017.12.016 PMC6733417

[3] R. L. Converse , T. N. Jacobsen , R. D. Toto , C. M. T. Jost , F. Cosentino , F. Fouad‐Tarazi , R. G. Victor , N. Engl. J. Med. 1992, 327, 1912. 1454086 10.1056/NEJM199212313272704

[4] R. Veelken , R. E. Schmieder , Nat. Rev. Nephrol. 2014, 10, 305. 24733118 10.1038/nrneph.2014.59

[5] C. Zoccali , F. Mallamaci , G. Tripepi , S. Parlongo , S. Cutrupi , F. A. Benedetto , A. Cataliotti , L. S. Malatino , Hypertension 2002, 40, 41. 12105136 10.1161/01.hyp.0000022063.50739.60

[6] M. R. Noh , H. S. Jang , J. Kim , B. J. Padanilam , Int. J. Mol. Sci. 2020, 21, 1647. 32121260 10.3390/ijms21051647 PMC7084190

[7] J. Kim , B. J. Padanilam , J. Am. Soc. Nephrol. 2013, 24, 229. 23264683 10.1681/ASN.2012070678 PMC3559485

[8] J. Kim , B. J. Padanilam , Kidney Int. 2015, 87, 350. 25207878 10.1038/ki.2014.300 PMC4312521

[9] H. S. Jang , J. Kim , B. J. Padanilam , Kidney Res. Clin. Pract. 2019, 38, 6. 30831675 10.23876/j.krcp.18.0143 PMC6481969

[10] M. D. Okusa , D. L. Rosin , K. J. Tracey , Nat. Rev. Nephrol. 2017, 13, 669. 28970585 10.1038/nrneph.2017.132 PMC6049817

[11] R. M. Touyz , Circ. Res. 2015, 117, 487. 26316603 10.1161/CIRCRESAHA.115.307176

[12] P. M. Tang , D. J. Nikolic‐Paterson , H. Y. Lan , Nat. Rev. Nephrol. 2019, 15, 144. 30692665 10.1038/s41581-019-0110-2

[13] X.‐M. Meng , S. Wang , X.‐R. u. Huang , C. Yang , J. Xiao , Y. Zhang , K. a‐F. To , D. J. Nikolic‐Paterson , H.‐Y. Lan , Cell Death Dis. 2016, 7, 2495. 10.1038/cddis.2016.402 PMC5261004 27906172

[14] M. B. Buechler , W. Fu , S. J. Turley , Immunity 2021, 54, 903. 33979587 10.1016/j.immuni.2021.04.021

[15] J. Guiot , M. Cambier , A. Boeckx , M. Henket , O. Nivelles , F. Gester , E. Louis , M. Malaise , F. Dequiedt , R. Louis , I. Struman , M.‐S. Njock , Thorax 2020, 75, 870. 32759383 10.1136/thoraxjnl-2019-214077 PMC7509395

[16] M. Y. Yao , W. H. Zhang , W. T. Ma , Q. H. Liu , L. H. Xing , G. F. Zhao , Exp. Mol. Med. 2019, 51, 1. 10.1038/s12276-019-0255-x PMC6547742 31164635

[17] L. Chen , X. Yao , H. Yao , Q. Ji , G. Ding , X. Liu , FASEB J. 2020, 34, 5178. 32061112 10.1096/fj.201902307RRR

[18] L. Chen , Y. Huang , Z. Duan , P. Huang , H. Yao , Y. u. Zhou , Q. Ji , X. Liu , Front. Cell Dev. Biol. 2021, 9, 716209. 34676206 10.3389/fcell.2021.716209 PMC8525629

[19] P. K. Govindappa , M. Patil , V. N. S. Garikipati , S. K. Verma , S. Saheera , G. Narasimhan , W. Zhu , R. Kishore , J. Zhang , P. Krishnamurthy , FASEB J. 2020, 34, 2238. 31907992 10.1096/fj.201901995R PMC8286699

[20] Y. Wang , M. Zhao , S. Liu , J. Guo , Y. Lu , J. Cheng , J. Liu , Cell Death Dis. 2020, 11, 924. 33116121 10.1038/s41419-020-03127-z PMC7595091

[21] Q. J. Zhu , M. Zhu , X. X. Xu , X. M. Meng , Y. G. Wu , FASEB J. 2019, 33, 9279. 31162940 10.1096/fj.201802427RRR

[22] X. Shan , C. Zhang , C. Mai , X. Hu , N. Cheng , W. Chen , D. Peng , L. Wang , Z. Ji , Y. Xie , Front. Mol. Biosci. 2021, 8, 715461. 34368234 10.3389/fmolb.2021.715461 PMC8333870

[23] S. Leach , K. Suzuki , Front. Immunol. 2020, 11, 1235. 32714319 10.3389/fimmu.2020.01235 PMC7344327

[24] E. P. Bottinger , M. Bitzer , J. Am. Soc. Nephrol. 2002, 13, 2600. 12239251 10.1097/01.asn.0000033611.79556.ae

[25] P. Minoo , L. Hu , N. Zhu , Z. Borok , S. Bellusci , J. Groffen , D. Kardassis , C. Li , Nucleic Acids Res. 2008, 36, 179. 18003659 10.1093/nar/gkm871 PMC2248754

[26] M. Fukuchi , T. Imamura , T. Chiba , T. Ebisawa , M. Kawabata , K. Tanaka , K. Miyazono , Mol. Biol. Cell 2001, 12, 1431. 11359933 10.1091/mbc.12.5.1431 PMC34595

[27] Z. Liu , E. Brunskill , B. Varnum‐Finney , C. Zhang , A. Zhang , P. Y. Jay , I. Bernstein , M. Morimoto , R. Kopan , Development 2015, 142, 2452. 26062937 10.1242/dev.125492 PMC4510869

[28] Q. Li , Y. Deng , L. Liu , C. Zhang , Y. Cai , T. Zhang , M. Han , G. Xu , Front. Immunol. 2021, 12, 823935. 35140713 10.3389/fimmu.2021.823935 PMC8818683

[29] S. Tanaka , M. D. Okusa , Kidney Int. 2020, 97, 466. 32001065 10.1016/j.kint.2019.10.032 PMC7039752

[30] X. Qin , X. Lin , L. Liu , Y. Li , X. Li , Z. Deng , H. Chen , H. Chen , Z. Niu , Z. Li , Y. Hu , J. Cell. Mol. Med. 2021, 25, 4466. 33834616 10.1111/jcmm.16524 PMC8093963

[31] B. Shu , R. Z. Zhang , Y. X. Zhou , C. He , X. Yang , Cell Death Discov. 2022, 8, 266. 35585044 10.1038/s41420-022-01036-y PMC9117676

[32] H. Ding , L. X. Li , P. C. Harris , J. Yang , X. Li , Nat. Commun. 2021, 12, 4548. 34315885 10.1038/s41467-021-24799-x PMC8316472

[33] X. i. Liu , J. Miao , C. Wang , S. Zhou , S. Chen , Q. Ren , X. Hong , Y. Wang , F. F. Hou , L. Zhou , Y. Liu , Kidney Int. 2020, 97, 1181. 32139089 10.1016/j.kint.2019.11.026

[34] L. Birtwistle , X. M. Chen , C. Pollock , Int. J. Mol. Sci. 2021, 22, 6596. 34202940 10.3390/ijms22126596 PMC8235408

[35] H. Jing , S. Tang , S. Lin , M. Liao , H. Chen , J. Zhou , Cell Death Dis. 2019, 10, 367. 31068572 10.1038/s41419-019-1605-2 PMC6506498

[36] Q. Cao , D. C. Harris , Y. Wang , Physiology 2015, 30, 183. 25933819 10.1152/physiol.00046.2014

[37] H. M. Shen , L. X. Sha , J. L. Kennedy , D. W. Ou , Int. J. Immunopharmacol. 1994, 16, 905. 7868295 10.1016/0192-0561(94)90045-0

[38] R. N. Spengler , S. W. Chensue , D. A. Giacherio , N. Blenk , S. L. Kunkel , J. Immunol. 1994, 152, 3024. 8144901

[39] M. P. Bebelman , C. Crudden , D. M. Pegtel , M. J. Smit , Trends Pharmacol. Sci. 2020, 41, 627. 32711926 10.1016/j.tips.2020.07.001

[40] M. Edeling , G. Ragi , S. Huang , H. Pavenstadt , K. Susztak , Nat. Rev. Nephrol. 2016, 12, 426. 27140856 10.1038/nrneph.2016.54 PMC5529143

[41] P. F. Christopoulos , T. T. Gjolberg , S. Kruger , G. Haraldsen , J. T. Andersen , E. Sundlisaeter , Front. Immunol. 2021, 12, 668207. 33912195 10.3389/fimmu.2021.668207 PMC8071949

[42] T. Niranjan , B. Bielesz , A. Gruenwald , M. P. Ponda , J. B. Kopp , D. B. Thomas , K. Susztak , Nat. Med. 2008, 14, 290. 18311147 10.1038/nm1731

[43] M. Huang , J. Zhang , H. Xu , T. Ding , D. Tang , Q. Yuan , L. Tao , Z. Ye , Cell Signal 2018, 51, 139. 30081092 10.1016/j.cellsig.2018.08.002

[44] S. Djudjaj , C. Chatziantoniou , U. Raffetseder , D. Guerrot , J.‐C. Dussaule , P. Boor , M. Kerroch , L. Hanssen , S. Brandt , A. Dittrich , T. Ostendorf , J. Floege , C. Zhu , M. Lindenmeyer , C. D. Cohen , P. R. Mertens , J. Pathol. 2012, 228, 286. 22806125 10.1002/path.4076

[45] T. D. Cummins , M. D. Mendenhall , M. N. Lowry , E. A. Korte , M. T. Barati , S. J. Khundmiri , S. A. Salyer , J. B. Klein , D. W. Powell , Biochim. Biophys. Acta 2011, 1814, 1748. 22001063 10.1016/j.bbapap.2011.09.010 PMC3223271

[46] R. Huang , Q. Zhou , P. Veeraragoo , H. Yu , Z. Xiao , Ren Fail 2011, 33, 207. 21332343 10.3109/0886022X.2011.553979

[47] Z. Xiao , J. Zhang , X. Peng , Y. Dong , L. Jia , H. Li , J. Du , Int. J. Biochem. Cell Biol. 2014, 55, 65. 25150830 10.1016/j.biocel.2014.08.009

[48] B. Zhou , W. Lin , Y. Long , Y. Yang , H. Zhang , K. Wu , Q. Chu , Signal Transduct. Target Ther. 2022, 7, 95. 35332121 10.1038/s41392-022-00934-y PMC8948217

[49] Q. Wang , Q. Lu , Nat. Commun. 2017, 8, 709. 28955033 10.1038/s41467-017-00767-2 PMC5617834

[50] K. Wei , I. Korsunsky , J. L. Marshall , A. Gao , G. F. M. Watts , T. Major , A. P. Croft , J. Watts , P. E. Blazar , J. K. Lange , T. S. Thornhill , A. Filer , K. Raza , L. T. Donlin , J. Albrecht , J. H. Anolik , W. Apruzzese , B. F. Boyce , D. L. Boyle , S. L. Bridges , J. H. Buckner , V. P. Bykerk , E. DiCarlo , J. Dolan , T. M. Eisenhaure , G. S. Firestein , C. Y. Fonseka , S. M. Goodman , E. M. Gravallese , P. K. Gregersen , et al., Nature 2020, 582, 259. 32499639 10.1038/s41586-020-2222-z PMC7841716

[51] B. Hu , Z. Wu , D. Bai , T. Liu , M. R. Ullenbruch , S. H. Phan , Am. J. Pathol. 2015, 185, 3066. 26358219 10.1016/j.ajpath.2015.07.014 PMC4630167

[52] B. Bielesz , Y. Sirin , H. Si , T. Niranjan , A. Gruenwald , S. Ahn , H. Kato , J. Pullman , M. Gessler , V. H. Haase , K. Susztak , J. Clin. Invest. 2010, 120, 4040. 20978353 10.1172/JCI43025 PMC2964979

[53] L. Hering , M. Rahman , S. A. Potthoff , L. C. Rump , J. Stegbauer , Front. Physiol. 2020, 11, 566871. 33240096 10.3389/fphys.2020.566871 PMC7680782

[54] M. Kretzler , L. Allred , Nat. Med. 2008, 14, 246. 18323843 10.1038/nm0308-246

[55] W. Wu , X. Wang , X. Yu , H. Y. Lan , Int. J. Biol. Sci. 2022, 18, 2795. 35541902 10.7150/ijbs.71595 PMC9066101

