# Supplementary material for: Sympathetic Activation Promotes Kidney Fibrosis in Mice via Macrophage‐Derived N2ICD‐Enriched Extracellular Vesicles
Source: Adv Sci (Weinh). 2025 Sep 4;12(44):e04607. doi: 10.1002/advs.202504607 (PMC12667535; doi:10.1002/advs.202504607)
Supplement: Supplementary file 1 — Supporting Information [file ADVS-12-e04607-s002.docx]

**Supplementary Material and Method**

**This PDF file includes:**

**1. Supplementary Methods**

**2. Supplementary Figures**

**3. Supplementary References**

**Supplementary Methods**

**Reagents**

Norepinephrine (HY-13715A), GW4869 (HY-19363), MG132 (HY-13259), polybrene (HY-112735), and puromycin (HY-B1743A) were purchased from MedChemExpress. TGF-β1 (240-B-002) was purchased from R&D system. Diphtheria toxin (DT) (D0564) was purchased from Sigma-Aldrich.

**Mice**

Male C57BL/6 mice (6-8-weeks-old) were used for all experiments. Notch2^F/F^ mice were purchased from the Jackson Laboratory (catalog number:010525). Pdgfrb^Cre^ (Quote: TGP221121YY1) and LysM^Cre^ (Quote: C001003) mice were obtained from the Cyagen Biosciences Inc. (Suzhou, China). Notch2^F/F^ mice crossed with LysM^Cre^ and Pdgfrb^Cre^ mice to generate Notch2^F/F^LysM^Cre^(M-Notch2^KO^) and Notch2^F/F^Pdgfrb^Cre^(F-Notch2^KO^), respectively. M-Notch2^KO^ and F-Notch2^KO^ mice were crossed to generate Notch2^F/F^LysM^Cre^ /Pdgfrb^Cre^ mice (M/F-Notch2^KO^). CD68^Cre^ mice were generated using the CRISPR/Cas9 strategy at the Shanghai Model Organisms Center, Inc. (NO. NM-KI-200192, Shanghai, China). CD68^Cre^ mice were crossed with Rosa26^tdtomato^ mice[1] to obtain mice with tdTomato-labelled macrophages (CD68^Cre^Rosa26^tdtomato^). Rosa26^iDTR^ mice[2] were crossed with Rosa26^iGFP^ mice[3] to obtain Rosa26^iDTR^Rosa26^iGFP^ mice, and then were mated with LysM^Cre^ mice to generate LysM^Cre^/Rosa26^iDTR^Rosa26^iGFP^ mice. α2B-AR^F/F^ mice were generated using the CRISPR/Cas9 strategy at the Shanghai Model Organisms Center, Inc. (NO. N1-3403, Shanghai, China). Macrophage-specific α2B-AR knockout mice (M-α2B^KO^ mice) were generated by crossbreeding LysM^Cre^ with α2B-AR^F/F^ mice. The floxed NICD2 transgenic (R26^N2ICD^) mice were generated using the CRISPR/Cas9 strategy at the Cyagen Biosciences Inc. (Quote: KICMS180607AN1, Suzhou, China) and crossed with Pdgfrb^Cre^ mice to obtain fibroblast-specific N2ICD overexpressed mice (F-R26^N2ICD^). Mice were maintained in the specific pathogen free animal laboratory of Tianjin Medical University in an environment with controlled temperature (22 °C ± 1 °C) and relative humidity (50% ± 5%) on a 12:12 h light/dark cycle, with free access to sterile food and water. All animal experiments were performed in accordance with the approval of the Laboratory Animal Management and Use Committee of the Tianjin Medical University. Humane end points and steps were performed to minimize animal suffering according to the guidelines.

Sample size was determined by power analysis based on our previous experiments.[4, 5] Animals were assigned a numerical code to ensure that experiments and analyses were performed in a blinded manner and were randomly assigned to either the experimental treatment group or the control group (using random table). All analyses were performed in a blinded fashion way: animals’ experimental conditions were unknown by the operator during measurement and data collection. All mice were included in the analysis, unless there was clear evidence of technical failures, such as malfunctioning equipment.

**Murine models of kidney fibrosis**

The mice were anesthetized by isoflurane inhalation. For the unilateral nephrectomy plus contralateral ischemia-reperfusion injury (Npx-IRI)-induced kidney fibrosis,[6] the renal pedicle was exposed by flank incision, and the right renal pedicle was clamped for 30 min, and the left kidney was removed. For reperfusion, the clamp was released and the kidney was monitored for color change to confirm blood reflow before suturing. In the sham group, the animals only underwent dorsal incision and exposure of the renal pedicle. The kidneys and plasma of IRI mice were harvested 21 days after Npx-IRI injury.

For the repeated low-dose cisplatin (RLDC) model,[7] 8 mg/kg body weight cisplatin (1.0 mg/ml solution in sterile 0.9% saline) or vehicle (sterile 0.9% saline) were administered intraperitoneally once a week for 24 days. Kidneys were harvested after injury induction for further analysis.

Renal denervation was performed 2 days before Npx-IRI, as previously described.[8] Briefly, the left renal artery and vein were exposed through the abdominal incision and isolated from the surrounding connective tissue. For nerve stripping, the renal vessels were painted with 95% ethanol for 2 minutes and then with PBS for 2 minutes. Control mice were subjected to the same surgical procedure without stripping of the kidney nerves.

**Kidney norepinephrine quantification​**

Norepinephrine (NE) levels in renal tissues were quantified using a commercial ELISA kit (Mouse NE ELISA Kit, Solarbio, Beijing, China; Cat# SEKSM-0019). Kidney tissues were homogenized in ice-cold PBS (1:10 w/v) using a mechanical homogenizer. Homogenates were centrifuged at 12,000×g for 15 min at 4°C. Supernatants were collected and NE concentrations were determined according to manufacturer protocols. Values were expressed as pg NE/mg kidney.

**Measurement of blood creatinine and urea nitrogen**

Plasma samples were collected from the Npx-IRI and RLDC mice. blood creatinine concentrations were measured using a commercial kit (C011-2-1, NJJC Bio, Nanjing, China), and blood urea nitrogen concentrations were measured using a quantitative colorimetric urea determination kit (DIUR-500, QuantiChrom urea assay kit, Hayward, CA, US), following manufacturer’s instructions.

**Histological analysis**

Mouse kidney samples were fixed in 4% paraformaldehyde. Paraffin sections of 5-μm thickness were sliced. The sections were dewaxed and hydrated for hematoxylin and eosin (H&E) staining. Sirius red staining was performed using a commercial kit (S8060, Solarbio) according to the standard procedures. Tissue fibrosis was calculated as a percentage of collagen content (Sirius Red positive area/total area) by digital image analysis using Image-Pro Plus 4.5 software[9] (Media Cybernetics, Silver Spring, USA).

For immunofluorescence staining, glass coverslips with cells or kidney sections were fixed and treated with 0.3% Triton X-100 in phosphate-buffered saline (PBS) for 30 min for permeabilization and blocked with 5% goat serum for 60 min. The sections were then incubated with Fsp1 (1:500, ab9328, Abcam) and Notch2 (1:1000, 5732, Cell Signaling Technology) at 4 °C overnight. The samples were then washed with PBS and incubated with secondary antibodies (Invitrogen) for 2 h at room temperature. As negative controls, species- and isotype-matched IgGs were used instead of primary antibodies. ProLong Gold antifade reagent with DAPI (Invitrogen) was used to mount and counter-stain specimens. Immunofluorescence images were captured using a Zeiss laser-scanning confocal microscope[10].

The tubular injury score was evaluated based on the degree of tubular necrosis, cast formation, tubular dilatation, and brush border loss as previously reported.[11] At least 10 fields (×200 magnification) in the maximal longitudinal cross-section of each mouse kidney were examined.

**Peritoneal macrophage isolation**

Peritoneal macrophages were induced via an intraperitoneal injection of 3% Brewer’s thioglycolate.[10] Three days later, the mice were euthanized, and 10 ml PBS was injected into the peritoneal cavity. The injected fluid was collected after a gentle massage of the peritoneum. The collected fluid was centrifuged at 300 × g for 10 min and the supernatant was discarded. The macrophages were resuspended in RPMI-1640 medium (C11875500BT, Thermo Fisher Scientific, Suzhou, China), supplemented with 10% fetal bovine serum (10270, Life Technologies, South America)，100 U/ml penicillin, and 100 μg/ml streptomycin (Life Technologies) and allowed to adhere at 37 °C overnight under 5% CO_2_. Any unattached cells were removed by washing with fresh medium before use.

**Primary renal fibroblasts isolation**

Kidney fibroblasts were isolated as described previously.[12] The cortices were removed and minced into 1-mm^2^ sections on ice. The minced tissues were transferred into a 1% gelatin-coated dish and cultured overnight in DMEM supplemented with 20% FBS, 100 U/ml penicillin and 100 μg/ml streptomycin. Kidney fibroblast cells were harvested when primary fibroblasts crawled out of tissue explants.

**Macrophages and renal fibroblast co-culture system**

To investigate the cell-cell communication between macrophages and fibroblasts, co-culture systems were established as described previously.[13] Primary mouse macrophages were seeded at a density of 1 × 10^7^ cells/well in Transwell plates (0.4-μm pore size) (3450, Costar, ME, USA). NRK-49F (CRL-1570, American Type Culture Collection, Manassas, VA, USA) cells at a density of 1 × 10^6^ cells/well, a gift from Prof. Dai Chunsun (Nanjing Medical University) were added simultaneously to the lower compartment and cultured in Dulbecco’s modified Eagle’s medium/F-12 medium (DMEM/F-12, C11330500BT, Thermo Fisher Scientific, Suzhou, China) supplemented with 5% fetal bovine serum, 10 ng/ml TGF-β, 100 U/ml penicillin, and 100 μg/ml streptomycin. Macrophages were stimulated with 10 µM of NE or DMSO. To block the excretion of EVs, 10 μM GW4869 was added to the macrophages for 6 h before treatment with NE. Macrophages and fibroblasts were co-cultured for 12 h before immunofluorescence evaluation of EV delivery or 48 h before detection of fibrogenic genes in NRK-49F cells.

**EV isolation, purification, and analysis**

Macrophages were treated with or without 10 µM NE for three days. The culture medium was centrifuged at 1000 × g for 10 min and filtrated through a 0.45 μm filter to eliminate the cells and debris. The culture medium was then ultracentrifuged at 200,000 × g for 120 min (Type 70 Ti rotor; Beckman Coulter Optima L-80 XP). The EV pellet was washed with PBS and collected by ultracentrifugation at 200,000 × g for 120 min. Purified EVs were confirmed by immunoblot analysis of surface markers (Alix, CD63, and TSG101). Size distribution, morphology, and quantity were determined using electron microscopy and nanoparticle tracking analysis (NTA).

**LC-MS/MS-based proteomics**

EVs were lysed by SDT (4% (w/v) SDS, 100mM Tris/HCl pH 7.6, 0.1 M DTT). The proteins were extracted and quantified using the BCA method and then digested using the filter-aided proteome preparation method, and the peptides were desalted using C18 Cartridge, lyophilized, and quantified (OD280). LC-MS/MS analysis was performed using a Q Exactive mass spectrometer (Thermo Fisher Scientific, Waltham, MA, USA) coupled to an Easy nLC (Thermo Fisher Scientific), as previously described.[14] The MS data were analyzed using MaxQuant software version 1.5.3.17 (Max Planck Institute of Biochemistry, Martinsried, Germany). The MASCOT engine (Matrix Science, London, UK; version 2.2) was used to search for MS/MS spectra embedded into Proteome Discoverer 1.4 (Thermo Electron, San Jose, CA.).

**RT-PCR**

Total RNA was extracted using TRIzol reagent (15596026, Invitrogen). Total RNA (1 μg) was reverse-transcribed to cDNA using a Reverse Transcription Reagent Kit (RR047A, Takara Bio Inc., Beijing, China). The resulting cDNA was amplified as previously described.[9] Each sample was analyzed twice and its expression was normalized to the internal control. The sequences of the primers used for RT-PCR are presented in Supplemental Table 1.

**Flow cytometric analysis**

Spleen samples were collected and ground through a 70-μm filter on ice. The cells were incubated with 1% BSA in PBS containing primary antibodies for 0.5h at 4 °C. The primary antibodies were diluted as follows: FITC-CD3 (1:200, 100203; BioLegend), PE/cy7-NK1.1 (1:200, 108714, BioLegend), APC-CD45 (1:200, 103116; BioLegend), PercCP/Cy5.5-CD19 (1:200, 152405; BioLegend), Bv421-F4/80 (1:200, 123131; BioLegend), and APC-CD11b (1:200, 101212; BioLegend). The cells were then washed twice before analysis, and sorting was performed to obtain macrophages using a BD FACSAria™ II flow cytometry system (BD Biosciences, San Jose, USA).

Macrophages in mouse kidneys were analyzed using a BD LSRFortessa™ cell analyzer (BD Biosciences, CA, USA). The kidney was gently minced in a glass Petri dish on ice and cut into small pieces. The tissue was then suspended in DMEM containing 0.1% collagenase (type IV, C4-BIOC, Sigma, Shanghai, China). The suspension was incubated in a 37 °C water bath for 30 min. After digestion, the suspension was strained through a 70-μm brass sieve and centrifuged for 10 min at 1,000 × g. The pellet was resuspended in the same solution, but without collagenase, and centrifuged again for 10 min. The cells were incubated with the following diluted primary antibodies: Bv421-F4/80 (1:200, 123131, BioLegend), APC-CD11b (1:200, 101212, BioLegend), APC-CD45 (1:200, 103116, BioLegend), and PE/cy7 Ly-6G (1:200, 560601, BD). The cells were washed twice before the analysis. The final data were analyzed using the FlowJo software (v.9; Tree Star).

**siRNA transfection**

Macrophages were transfected with siRNA using INTERFERin® polypus (Polyplus-transfection) for 24 h before being used for further analysis, according to the manufacturer’s protocol. All siRNA sequences were designed and produced by GenePharma (Suzhou, China): α1A-AR-sense: GAUACAUUGGUGUGAGCUA; antisense: UAGCUCACACCAAUGUAUC; α2B-AR-sense: UGAAGAUGGUGUAGAUGACAG; antisense: GUCAUCUACACCAUCUUCAAC; β2-AR-sense: CAAGUUCGAGCGACUACAA; antisense: UUGUAGCGCUCGAACUUG.

**Plasmid construction and transfection**

The plasmids were generated by Hanbio Biotechnology (Shanghai, China). cDNAs encoding N2ICD, N△P, N△A, and N△R with 3×Flag, Smad3, S△NL, and S△C with 3×HA, and ROC1 with 3×Myc were cloned into the pCDH-CMV-MCS-EF1-GFP-T2A-Puro vector(pCDH). The correct insertion of DNA fragments was confirmed by sequencing. All plasmids were transfected into NIH 3T3 cells using a transfection reagent (FT231-02, TransIntro) according to the manufacturer’s protocol.

**Lentivirus generation and transduction**

To generate lentiviruses, pCDH-N2ICD or pCDH-Smad3 was co-transfected with psPAX2 and pMD2.G into HEK 293T cells at a ratio of 4:3:1, using a transfection reagent (FT231-02, TransIntro) according to the manufacturer’s instructions. Cells were maintained for 16 h after transfection and cultured for another 48 h after changing the medium. The conditioned medium was collected and centrifuged to remove cell debris, and then filtered through a 0.45-μm pore size filter (HVLP04700, Durapore® Millipore). Transduction was achieved by mixing 1 ml virus, 1 ml fresh medium with NRK-49F cells, and 1 μg/ml polybrene in six-well plates. For lentiviral plasmids containing the puromycin resistance gene, medium containing 1 μg/ml puromycin was added to the cells at passage one. GFP proteins were examined by fluorescence microscopy to confirm the transduction efficiency. Passages two and three were considered early stable pools.

**Co-immunoprecipitation and western blotting**

The samples were homogenized in lysis buffer (P0013C, Beyotime) containing a protease inhibitor cocktail (Roche Diagnostics, Mannheim, Germany). The protein concentration in the lysis buffer was measured using the modified Lowry method (Bio-Rad DC protein assay reagent, Bio-Rad). For co-immunoprecipitation, cell lysates were incubated with the indicated antibodies for 12 h, followed by protein A/G beads for another 3 h, washed, and eluted with sample buffer (Bio-Rad). Proteins were size-separated by sodium dodecyl sulfate–polyacrylamide gel electrophoresis (SDS-PAGE) on Laemmli gels and then electroblotted onto polyvinylidene difluoride (PVDF) membranes. The PVDF membranes were blocked for 1 h with 5% nonfat dry milk before overnight incubation with primary antibodies as follow: Alix (1:1000, 2171, Cell Signaling Technology), TSG101 (1:1000, ab125011, Abcam), CD63 (1:1000, AB59479, Abcam), Ncl (1:1000, 14574, Cell Signaling Technology), Wdr61 (1:1000, SAB1401852, sigma), Fibronectin (1:5000, ab2413, Abcam), CTGF (1:5000, ab6992, Abcam), α-SMA (1:1000, A7248, abclonal), Smad3 (1:2000, 9523, Cell Signaling Technology), p-Smad3 (1:1000, 9520, Cell Signaling Technology), Smad2 (1:2000, 5339, Cell Signaling Technology), p-Smad2 (1:1000, 3108, Cell Signaling Technology), Notch2 (1:1000, 5732, Cell Signaling Technology), HA-Tag (1:1000, 3724, Cell Signaling Technology), Myc-Tag (1:1000, 2272, Cell Signaling Technology), Flag-Tag (1:1000, 2368, Cell Signaling Technology), α-Tubulin (1:5000, 3873, Cell Signaling Technology), and GAPDH (1:5000, 10494-1-AP, proteintech). The attached primary antibodies were identified using horseradish peroxidase-labelled anti-mouse/rabbit IgG (1:5000, 7076/7074, Cell Signaling Technology). Blots were developed using an enhanced chemiluminescence reagent (Thermo Fisher Scientific). The relative protein density was quantified using ImageJ 1.44 software.[10]

**Chromatin Immunoprecipitation**

*Smad3* overexpressed NRK-49F cells were seeded in 15 cm dishes and grown to 70–80% confluence. A ChIP assay was performed using a Simple ChIP Enzymatic Chromatin IP Kit (9003, Cell Signaling Technology), according to the manufacturer’s protocol. Briefly, cells were fixed with 1% formaldehyde/PBS, and the reaction was stopped by the addition of glycine, followed by washing with PBS. The pellet was lysed and the chromatin was sheared into 200-1000-bp fragments by sonication. The chromatin extract was incubated with rabbit anti-HA tag antibody (1:50, 3724, Cell Signaling Technology) at 4 °C overnight, with rotation. The conjugated beads were washed and eluted, and the cross links were reversed by incubation in the presence of proteinase K at 65 °C for 4 h. After purification, the DNA was dissolved in elution buffer and used for qPCR analysis. The JASPAR 2018 CORE vertebrate collection of position frequency matrices (PFMs) was used to predict Smad3 binding sites in the rat genome. A match score with a *p*-value < 0.001 was considered a high-confidence binding-site prediction. PCR primers were designed to span individual CTGF, ACTA2, COL1A1, and COL2A1 binding motifs (Supplemental Table 2).

**Single cell RNA-seq data analysis**

We analyzed previously published scRNA-seq data from IRI mouse kidney (GSE 139107) and human adult kidney (GSE 134355), using the Seurat package (v4.1.1) in R (v4.2.1). In both mouse and human datasets, we discarded cells that contained less than 500 genes. We log-transformed and scaled the expression counts with the R function log and compared the distribution of Notch2 in the main cell types. The expression of Notch2 was quantified and visualized using a dot plot. The color of the nodes was determined by the average expression value of all cells that expressed Notch2, and the size of the nodes was determined by the percentage of cells expressing Notch2 in a cell type.

**Statistics**

All data are expressed as mean ± standard error of the mean (SEM). Data were analyzed using SPSS version 21.0 (IBM Inc., Armonk, NY, USA). The normal distribution of the data was examined using the Shapiro-Wilk normality test. Mann–Whitney U test was used to compare two independent samples. For comparisons of multiple groups, one-way or two-way ANOVA was used, followed by the *post hoc* Bonferroni test. Statistical significance was set at *p* < 0.05.

**Supplementary figure 1. The effects of GW4869 on EV formation.** (A) Size distribution and (B) average concentration of extracellular vesicles (EVs) secreted by NE-treated macrophages with or without 10 μM GW4869. Data are presented as mean ± standard error of the mean (SEM). Statistical significance was evaluated using two-way ANOVA.

**Supplementary figure 2. Generation of macrophage-specific Notch2-deleted mice.** A. Genotyping of macrophage-specific Notch2-deleted mice by PCR of genomic DNA extracted from the tail biopsies. B. Relative mRNA levels of Notch2 in T cells, B cells and macrophages from spleens of control and M-Notch2^KO^ mice. n = 4–6. Data represent mean ±SEM. Statistical significance was evaluated by Mann–Whitney U test (B). **, *p* < 0.01.

**Supplementary figure 3. Quantification of norepinephrine (NE) levels in the kidney of mice 21 days after Npx-IRI.** n=6. Data represent mean ± SEM. Statistical significance was evaluated by Mann–Whitney U test. ***, *p* < 0.001.

**Supplementary figure 4.** **Sympathetic denervation ameliorates Npx-IRI -induced renal fibrosis in mice**. A-B. H&E staining(*A*) and tubular injury score (*B*) of kidneys of renal denervated mice underwent Npx-IRI. Scale bar: 100 μm. C-D. Blood urea nitrogen (*C*) and creatinine (*D*) levels of Npx-IRI mice with renal denervation (DNx). n = 8. E-F. Relative mRNA levels of KIM-1 (E) and NGAL (F) in kidneys from renal denervated mice underwent Npx-IRI. n =8. G-H. Sirius red staining (*G*) of kidneys from renal denervated mice underwent Npx-IRI and its quantification (*H*). Scale bar: 100 μm; n = 8. I. Immunoblot analysis of CTGF, Fibronectin and α-SMA expression in kidneys from renal denervated mice underwent Npx-IRI. Data represent mean ±SEM. Statistical significance was evaluated by Mann–Whitney U tests (H) and Two-way ANOVA, followed by Tukey’s test for multiple comparisons (B, C, D, E, F). ***, *p* < 0.001.

**Supplementary figure 5. Depletion of macrophages using macrophage-specific diphtheria toxin receptor** **transgenic mice.** A. Scheme of targeted depletion of macrophages in mice subjected to Npx-IRI. B. Representative flow cytometric profiles of macrophages in kidneys from LysM^Cre^/Rosa26^iDTR^Rosa26^iGFP^ mice underwent Npx-IRI after DT administration. Sal: Saline, DT: Diphtheria toxin.

**Supplementary figure 6. Quantification of norepinephrine (NE) levels in the kidney of mice 24 days after RLDC treatment.** n=6. Data represent mean ± SEM. Statistical significance was evaluated by Mann–Whitney U test. ***, *p* < 0.001.

**Supplementary figure 7. Depletion of macrophages reduces RLDC-induced renal fibrosis in mice.** A-B. H&E staining (*A*) and tubular injury score (*B*) of kidneys from LysM^Cre^/Rosa26^iDTR^Rosa26^iGFP^ mice treated with RLDC after DT administration. Scale bar: 100 μm. C-D. Blood urea nitrogen (*C*) and creatinine (*D*) levels of LysM^Cre^/Rosa26^iDTR^Rosa26^iGFP^ mice treated with RLDC after DT administration. n = 8. E-F. Relative mRNA levels of KIM-1 (*E*) and NGAL (*F*) in kidneys from LysM^Cre^/Rosa26^iDTR^Rosa26^iGFP^ mice treated with RLDC after DT administration. n = 8. G-H. Sirius red staining (*G*) of kidneys from LysM^Cre^/Rosa26^iDTR^Rosa26^iGFP^ mice treated with RLDC after DT administration and its quantification (*H*). Scale bar: 100 μm; n = 8. I. Immunoblot analysis of CTGF, Fibronectin and α-SMA expression in kidneys from LysM^Cre^/Rosa26^iDTR^Rosa26^iGFP^ mice treated with RLDC after DT administration. Data represent mean ±SEM. Statistical significance was evaluated by Mann–Whitney U tests (H) and Two-way ANOVA, followed by Tukey’s test for multiple comparisons (B, C, D, E, F). ***, *p* < 0.001.

**Supplementary figure 8. Deletion of Notch2 in macrophages protects against RLDC-induced renal fibrosis in mice.** A-B. H&E staining (*A*) and tubular injury score (*B*) of kidneys from M-Notch2^KO^ mice administrated with RLDC. Scale bar: 100 μm. C-D. Blood urea nitrogen *(C*) and creatinine (*D*) levels of M-Notch2^KO^ mice administrated with RLDC. n = 8. E-F. Relative mRNA levels of KIM-1 (*E*) and NGAL (*F*) in kidneys from M-Notch2^KO^ mice administrated with RLDC. n = 8. G-H. Sirius red staining (*G*) of kidneys from M-Notch2^KO^ mice administrated with RLDC and its quantification (*H*). Scale bar: 100 μm; n = 8. I. Immunoblot analysis of CTGF, Fibronectin and α-SMA expression in kidneys from M-Notch2^KO^ mice administrated with RLDC. Data represent mean ±SEM. Statistical significance was evaluated by Mann–Whitney U tests (H) and Two-way ANOVA, followed by Tukey’s test for multiple comparisons (B, C, D, E, F). **, *p* < 0.01; ***, *p* < 0.001.

**Supplementary figure 9. Notch2 expression in renal fibroblasts is barely detected during the development of renal fibrosis.** A. The dot plot of Notch2 expression in cells from normal mouse kidneys. PTS: segment of proximal tubule; DCT: distal convoluted tubule; CNT: connecting tubule; Pod: podocytes; EC: endothelial cells; MΦ: macrophage; Fib: fibroblasts; Per: pericytes. B. The dot plot of Notch2 expression in cells from healthy human kidneys. MΦ: macrophage; Fib: fibroblasts; EC: endothelial cells; EpiC: epithelial cells. C. The dot plot of Notch2 expression in mouse renal fibroblasts after 2 days, 14 days and 6 weeks of IRI. D. Representative images from confocal fluorescence microscopy of Notch2 protein expression in renal fibroblasts (Fsp1^+^) in kidneys from LysM^Cre^/Rosa26^iDTR^Rosa26^iGFP^ mice underwent Npx-IRI after DT administration. Scale bar: 10 μm.

**Supplementary figure 10. Generation of macrophage(M) and fibroblast(F)-specific Notch2-deficient mice.** A. Breeding scheme for generation of M/F-Notch2^KO^ mice by crossing F-Notch2^KO^ with M-Notch2^KO^ mice. B. Genotyping of macrophage- and fibroblast- specific Notch2 deletion mice by PCR of genomic DNA extracted from the tail biopsies. C. Relative mRNA levels of Notch2 in renal fibroblasts (*right*) and splenic macrophages (*left*) isolated from M-Notch2^KO^, F-Notch2^KO^ and M/F-Notch2^KO^ mice, respectively. n = 7-8. Data represent mean ± SEM. Statistical significance was evaluated by One-way ANOVA, followed by Tukey’s test for multiple comparisons (C). ***, *p* < 0.001. n.s., no significance.

**Supplementary figure 11. Deletion of Notch2 in renal fibroblasts has no obvious effect on Npx-IRI-induced renal fibrosis in mice.** A-B. H&E staining (*A*) and tubular injury score (*B*) of kidneys from M-Notch2^KO^, F-Notch2^KO^ and M/F-Notch2^KO^ mice underwent Npx-IRI, respectively. Scale bar: 100 μm. C-D. Blood urea nitrogen (C) and creatinine (D) levels of M-Notch2^KO^, F-Notch2^KO^ and M/F-Notch2^KO^ mice underwent Npx-IRI, respectively. n = 8. E-F. Relative mRNA levels of KIM-1 (*E*) and NGAL (*F*) in kidneys from M-Notch2^KO^, F-Notch2^KO^ and M/F-Notch2^KO^ mice underwent Npx-IRI, respectively. n = 8. G-H. Sirius red staining (*G*) of kidneys from M-Notch2^KO^, F-Notch2^KO^ and M/F-Notch2^KO^ mice underwent Npx-IRI, respectively, and its quantification (*H*). Scale bar: 100 μm; n = 8. I. Immunoblot analysis of CTGF, Fibronectin and α-SMA expression in kidneys from M-Notch2^KO^, F-Notch2^KO^ and M/F-Notch2^KO^ mice underwent Npx-IRI, respectively. Data represent mean ± SEM. Statistical significance was evaluated by One-way ANOVA, followed by Tukey’s test for multiple comparisons (B, C, D, E, F, H). n.s., no significance.

**Supplementary figure 12. Generation of transgenic mice expressing N2ICD in fibroblasts.** A. The strategy of targeting N2ICD to the Rosa26 locus (R26^N2ICD^). R26^N2ICD/+^ Pdgfrb^Cre^ mice (F-R26^N2ICD/+^) were generated by crossing R26^N2ICD/+^ mice with Pdgfrb^Cre^ mice. B. Genotyping of N2ICD transgenic mice by PCR analysis of genomic DNA extracted from the tail biopsies. C. Representative images from confocal fluorescence microscopy of N2ICD, EGFP and Fsp1 staining in renal tissue from F-R26^N2ICD/+^ and control mice. Arrows indicate EGFP, N2ICD and Fsp1 triple positive cells. Scale bar: 20 μm.

**Supplementary figure 13. Overexpression of N2ICD in fibroblasts exacerbates Npx-IRI -induced renal fibrosis in mice**. A-B. H&E staining (*A*) and tubular injury score (*B*) of kidneys from F-R26^N2ICD/+^ mice with Npx-IRI treatment. Scale bar: 100 μm. C-D. Blood urea nitrogen (*C*) and creatinine (*D*) levels of F-R26^N2ICD/+^ mice subjected to Npx-IRI. n = 6-8. E-F. Relative mRNA levels of KIM-1 (E) and NGAL (F) in kidneys from F-R26^N2ICD/+^ mice with Npx-IRI treatment. n = 6-8. G-H. Sirius red staining (*G*) of kidneys from F-R26^N2ICD/+^ mice with Npx-IRI treatment and its quantification (*H*). Scale bar: 100 μm; n = 8. I. Immunoblot analysis of CTGF, Fibronectin and α-SMA expression in kidneys from F-R26^N2ICD/+^ mice with Npx-IRI treatment. Data represent mean ±SEM. Statistical significance was evaluated by Two-way ANOVA, followed by Tukey’s test for multiple comparisons (B, C, D, E, F, H). **, *p* < 0.01; ***, *p* < 0.001.

**Supplementary figure 14. Knockdown of α2B adrenoceptor in macrophages abolishes NE-exposed macrophage-mediated renal fibroblast activation.** A. Relative mRNA levels of adrenergic receptors in mouse peritoneal macrophages with or without NE treatment. n = 6­–8. B. Knockdown efficiency of α1A, α2B and β2 adrenoceptor siRNAs in mouse peritoneal macrophages. n = 4-6. C. Co-culture system for investigation of the effect of EVs from siRNA transfected peritoneal macrophages on the activation of NRK-49F cells. D. Effect of knockdown of α1A, α2B or β2 adrenoceptor in NE-exposed peritoneal macrophages on the expression of CTGF (*left*), Fibronectin (*middle*) and ACTA2 (*right*) in TGFβ1-treated NRK-49F cells in the co-culture system. n = 4. Data represent mean ± SEM. Statistical significance was evaluated by Mann–Whitney U tests (A, B, D). *, *p* < 0.05; **, *p* < 0.01; ***, *p* < 0.001.

**Supplementary figure 15. Generation of macrophage-specific α2B-AR-deleted mice.** A. Schematic diagram depicting the locations of guide RNAs targeting the α2B-AR locus. M-α2B^KO^ mice were generated by crossing α2B-AR^F/F^ mice with LysM^Cre^ mice. B. Genotyping macrophage specific α2B-AR deficient mice by PCR analysis of genomic DNA extracted from the tail biopsies. C. Quantification of α2B-AR expression in splenic T cells, B cells and macrophages from control and M-α2B^KO^ mice. n = 6. Data represent mean ±SEM. Statistical significance was evaluated by Mann–Whitney U tests (C). **, *p* < 0.01.

**Supplementary figure 16. Macrophage-specific α2B-AR deletion protects against RLDC-induced renal fibrosis in mice**. A-B. H&E staining (*A*) and tubular injury score (*B*) of kidneys from M-α2B^KO^ mice administrated with RLDC. Scale bar: 100 μm. C-D. Blood urea nitrogen (*C*) and creatinine (*D*) levels of M-α2B^KO^ mice administrated with RLDC. n = 8. E-F. Relative mRNA levels of KIM-1 (E) and NGAL (F) in kidneys from M-α2B^KO^ mice administrated with RLDC. n=8. G-H. Sirius red staining (*G*) of kidneys from M-α2B^KO^ mice administrated with RLDC and its quantification (H). Scale bar: 100 μm; n = 8. I. Immunoblot analysis of CTGF, Fibronectin and α-SMA expression in kidneys from M-α2B^KO^ mice administrated with RLDC. Data represent mean ±SEM. Statistical significance was evaluated by Mann–Whitney U tests (H) and Two-way ANOVA, followed by Tukey’s test for multiple comparisons (B, C, D, E, F). **, *p* < 0.01; ***, *p* < 0.001.

**Supplementary figure 17. Effect of N2ICD overexpression on mRNA levels of Smad2/3 in renal fibroblasts.** Relative mRNA levels of Smad2 and Smad3 in NRK-49F cells infected N2ICD-packaged lentivirus. n = 6. Data represent mean ± SEM. Statistical significance was evaluated by Mann–Whitney U tests. n.s., no significance.

**Supplemental Figure 18. Knockdown of Smad3 abrogates N2ICD-induced fibrogenesis in NRK-49F cells.** (A) Validation of knockdown efficiency of Smad3 siRNAs in NRK-49F cells. n = 6. (B) Relative mRNA expression levels of fibrogenic marker gene CTGF, Fibronectin, and ACTA2 in N2ICD-overexpressing NRK-49F cells treated with si-Smad3. n = 6. Data are presented as mean ± SEM. Statistical significance was assessed using Two-way ANOVA. *, *p* < 0.05; **, *p* < 0.01.

**Supplemental Table 1. Primers for RT-PCR analysis.**

| Genes | Forward primer (5’-3’) | Reverse primer (5’-3’) |
| --- | --- | --- |
| *Rat* |  |  |
| Fibronectin | CCTTTTCTCCTGTTGTGGCC | CTTCCTCGCTCAGTTCGTAC |
| CTGF | AGGAGTGGGTGTGTGATGAG | AGACGACTCTGCTTCTCCAG |
| ACTA2 | CATCATGCGTCTGGACTTGG | CCAGGGAAGAAGAGGAAGCA |
| GAPDH | GTATGACTCTACCCACGGCA | AAGACGCCAGTAGACTCCAC |
| *Mouse* |  |  |
| β-actin | GGCTGTATTCCCCTCCATCG | CCAGTTGGTAACAATGCCATGT |
| Fibronectin | TCCCGGGCAGAAAGTACATT | TTCAGGGAGGTTGAGCTCTG |
| CTGF | GTGAGTCCTTCCAAAGCAGC | TAGTTGGGTCTGGGCCAAAT |
| ACTA2 | AACACGGCATCATCACCAAC | ACCAGTTGTACGTCCAGAGG |
| KIM-1 | AGACTGGAATGGCACTGTGA | GGCAACCACGCTTAGAGATG |
| NGAL | ATGCACAGGTATCCTCAGGT | CTTCAGTTCAGGGGACAGCT |
| α1A-AR | GGACGTCTTATGCTGCACAG | CACGTATCCTGGCTCCTCAT |
| α1B-AR | GAACCCTTCTACGCCCTCTT | CTCAGGGTCAGCTCTTTGGA |
| α1D-AR | GCTCCTTCTACCTCCCCATG | TTGCTACTCTGTGTCCCTGG |
| α2A-AR | CCCTTTTCTTTGGCCAACGA | CAATGATGGCCTTGATGCGA |
| α2B-AR | TCGGCCATCACCTTTCTCAT | TGGCCAGAGAGAAAGGGATG |
| α2C-AR | CCTACTGGTACTTCGGGCAA | GAAGGAGATGACAGCGGAGA |
| β1-AR | GCTCATCGTTCTGCTCATCG | GCAGAAGAAGGAGCCGTACT |
| β2-AR | GAGCGACTACAAACCGTCAC | TGGTACTTGAAGGGCGATGT |
| β3-AR | TGCTAGCATCGAGACCTTGT | GCATGTTGGAGGCAAAGGAA |

**Supplemental Table 2. Primers for ChIP qPCR analysis.**

| Binding sites | Forward primer (5’-3’) | Reverse primer (5’-3’) |
| --- | --- | --- |
| ACTA2  -275 ~ -262  AGTGTC*T*GGGCATT | GACAGGAAGAGCTGGCATCT | ATTCCTCTGCTCTGCTCTCG |
| -356 ~ -343  AAAGGC*TA*GCCTGA | TGTTGCCTTAAAGTCCCAGC | ACAACTGCTCAAATGCCCAG |
| -1046~ -1033  GTGAG*TA*GACAGCT | TCAAAGACAGCTATGCGTGC | CTGCAAGCCAAGGTTCTGAT |
| CTGF  -56 ~ -43  CTTTGGC*G*AGCCGG | AGACGGAGGAATGTGGAGTG | CTGGCTTTTATACGCTCCGG |
| -1025 ~ -1012  TTACAGC*TA*GACAC | ACCGCTTTGCTGTCATGTAA | TGTCAGGGAACATAGGGTGG |
| -1076 ~ -1063  CACTTGT*A*GACACT | GAGAGAGAGAGAGAGAGAGAGAG | GCTTAGTCGGTGCCTTTGAT |
| COL1A1  -1979 ~ -1066  CCTACCCT*A*GACAA | CAACAAAGAGTGGGCAGATCA | AGTAAAGTCCTGCCTTCCACA |
| COL2A1  -935 ~ -922  CCACCTCC*A*GACTC | GGGATAGGGGAAGATGTGTGT | ACTGTTGACCACTCTGAGGC |
| -1310 ~ -1297  CTTCG*A*GACAAAGG | GGTAGCTTCTCCCAAGGACT | TTGCTCTGTTCCCGAAGTCT |

References

1. Jiang, Z., Feng, T., Lu, Z., et al., “PDGFRb(+) mesenchymal cells, but not NG2(+) mural cells, contribute to cardiac fat,” Cell Rep (2021): 34 (5), 108697, <https://doi.org/10.1016/j.celrep.2021.108697>.

2. Buch, T., Heppner, F.L., Tertilt, C., et al., “A Cre-inducible diphtheria toxin receptor mediates cell lineage ablation after toxin administration,” Nat Methods (2005): 2 (6), 419-26, <https://doi.org/10.1038/nmeth762>.

3. Liu, Q., Yang, R., Huang, X., et al., “Genetic lineage tracing identifies in situ Kit-expressing cardiomyocytes,” Cell Res (2016): 26 (1), 119-30, <https://doi.org/10.1038/cr.2015.143>.

4. He, Y., Zuo, C., Jia, D., et al., “Loss of DP1 Aggravates Vascular Remodeling in Pulmonary Arterial Hypertension via mTORC1 Signaling,” Am J Respir Crit Care Med (2020): 201 (10), 1263-76, <https://doi.org/10.1164/rccm.201911-2137OC>.

5. Kong, D., Wan, Q., Li, J., et al., “DP1 Activation Reverses Age-Related Hypertension Via NEDD4L-Mediated T-Bet Degradation in T Cells,” Circulation (2020): 141 (8), 655-66, <https://doi.org/10.1161/CIRCULATIONAHA.119.042532>.

6. Shi, M., Flores, B., Gillings, N., et al., “alphaKlotho Mitigates Progression of AKI to CKD through Activation of Autophagy,” J Am Soc Nephrol (2016): 27 (8), 2331-45, <https://doi.org/10.1681/ASN.2015060613>.

7. Fu, Y., Cai, J., Li, F., et al., “Chronic effects of repeated low-dose cisplatin treatment in mouse kidneys and renal tubular cells,” Am J Physiol Renal Physiol (2019): 317 (6), F1582-F92, <https://doi.org/10.1152/ajprenal.00385.2019>.

8. Kim, J., Padanilam, B.J., “Renal denervation prevents long-term sequelae of ischemic renal injury,” Kidney Int (2015): 87 (2), 350-8, <https://doi.org/10.1038/ki.2014.300>.

9. Ren, H., Zuo, S., Hou, Y., Shang, W., Liu, N., Yin, Z., “Inhibition of alpha1-adrenoceptor reduces TGF-beta1-induced epithelial-to-mesenchymal transition and attenuates UUO-induced renal fibrosis in mice,” FASEB J (2020): 34 (11), 14892-904, <https://doi.org/10.1096/fj.202000737RRR>.

10. Shang, W., Chen, G., Li, Y., et al., “Static Magnetic Field Accelerates Diabetic Wound Healing by Facilitating Resolution of Inflammation,” J Diabetes Res (2019): 2019, 5641271, <https://doi.org/10.1155/2019/5641271>.

11. Fang, W., Wang, Z., Li, Q., et al., “Gpr97 Exacerbates AKI by Mediating Sema3A Signaling,” J Am Soc Nephrol (2018): 29 (5), 1475-89, <https://doi.org/10.1681/ASN.2017080932>.

12. Grupp, C., Muller, G.A., “Renal fibroblast culture,” Exp Nephrol (1999): 7 (5-6), 377-85, <https://doi.org/10.1159/000020635>.

13. Vinas, J.L., Burger, D., Zimpelmann, J., et al., “Transfer of microRNA-486-5p from human endothelial colony forming cell-derived exosomes reduces ischemic kidney injury,” Kidney Int (2016): 90 (6), 1238-50, <https://doi.org/10.1016/j.kint.2016.07.015>.

14. Lu, T., Zhang, Z., Zhang, J., et al., “CD73 in small extracellular vesicles derived from HNSCC defines tumour-associated immunosuppression mediated by macrophages in the microenvironment,” J Extracell Vesicles (2022): 11 (5), e12218, <https://doi.org/10.1002/jev2.12218>.
