# Supplementary material for: Sympathetic Activation Promotes Kidney Fibrosis in Mice via Macrophage‐Derived N2ICD‐Enriched Extracellular Vesicles
Source: Adv Sci (Weinh). 2025 Sep 4;12(44):e04607. doi: 10.1002/advs.202504607 (PMC12667535; doi:10.1002/advs.202504607)

Supplementary Figure1

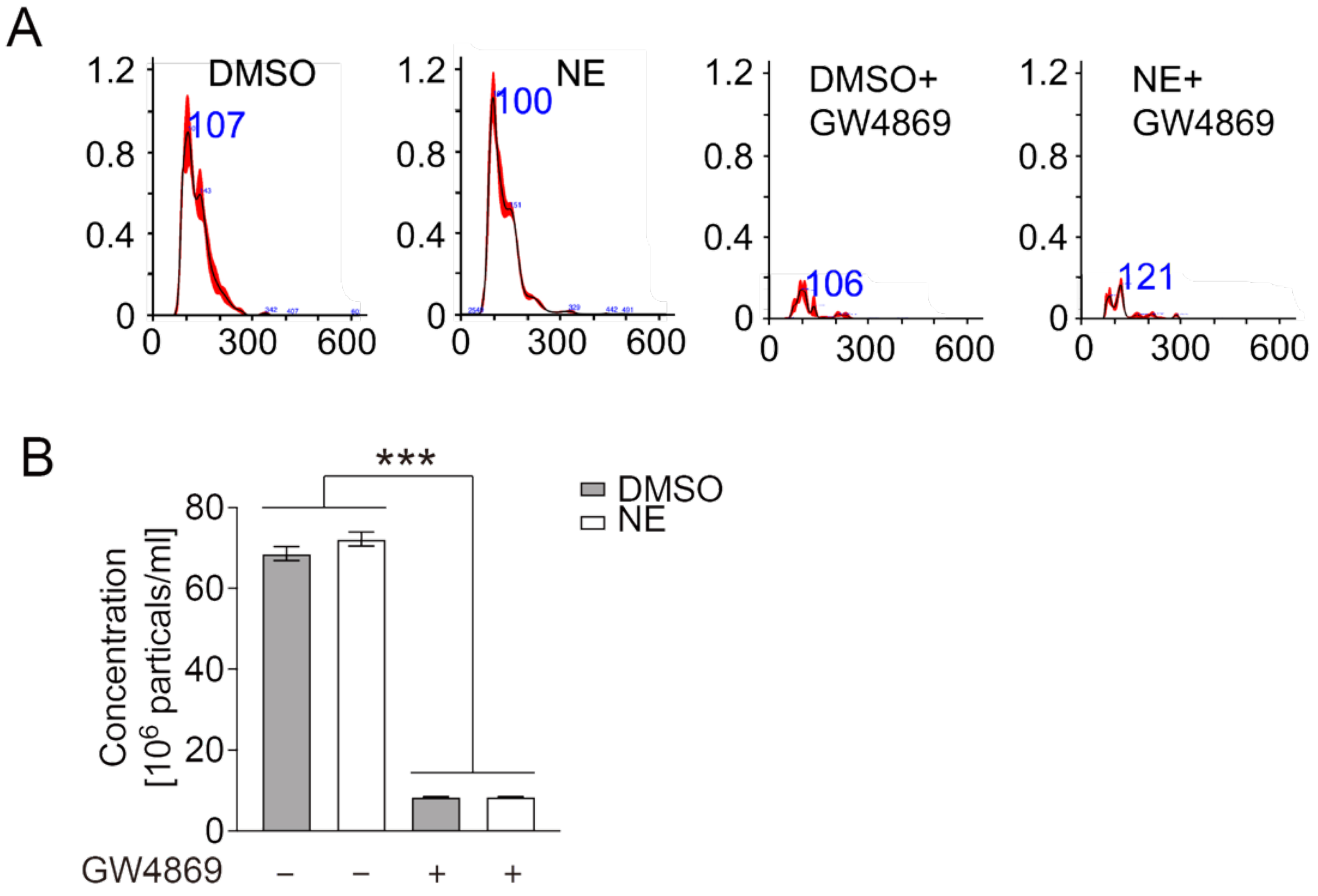

Supplementary Figure2

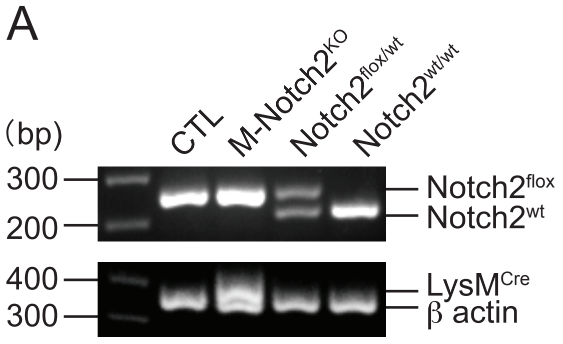

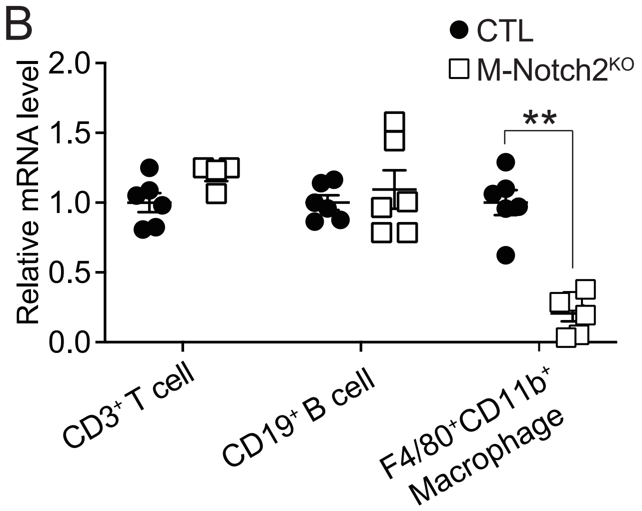

Supplementary Figure3

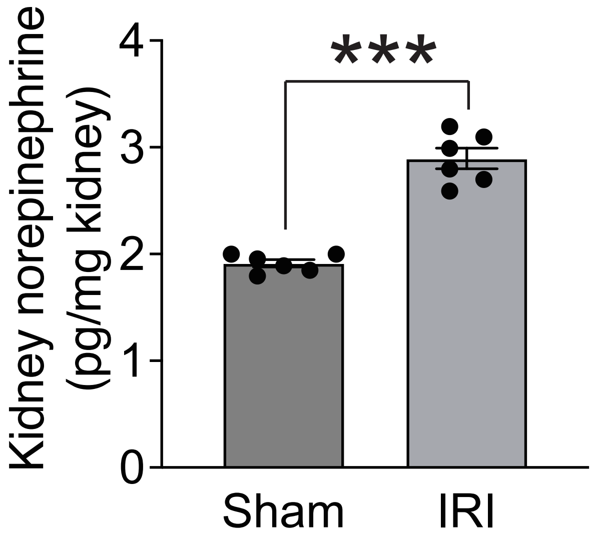

Supplementary Figure4

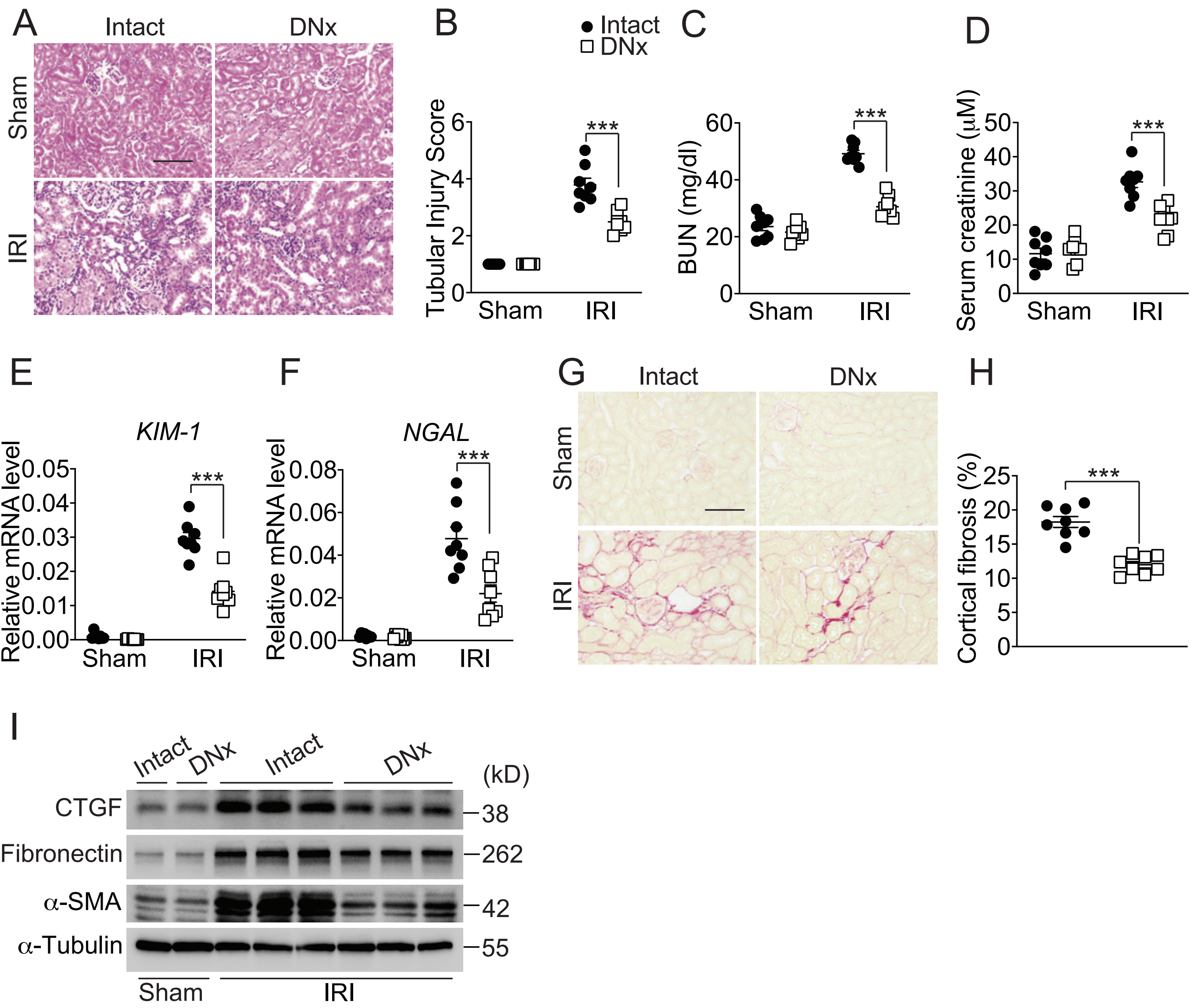

Supplementary Figure5

A

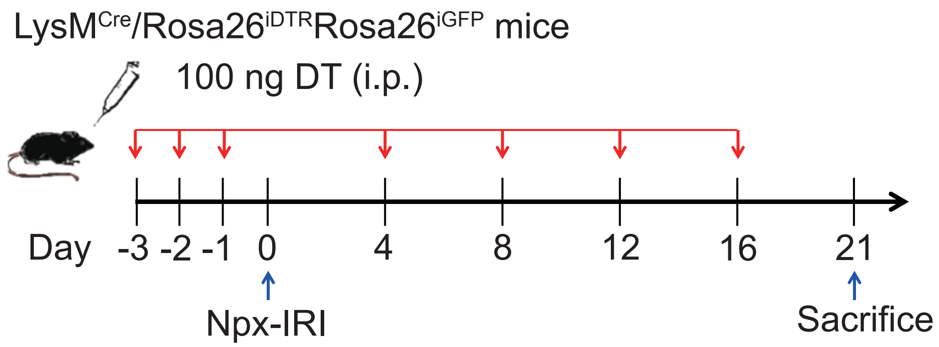

B

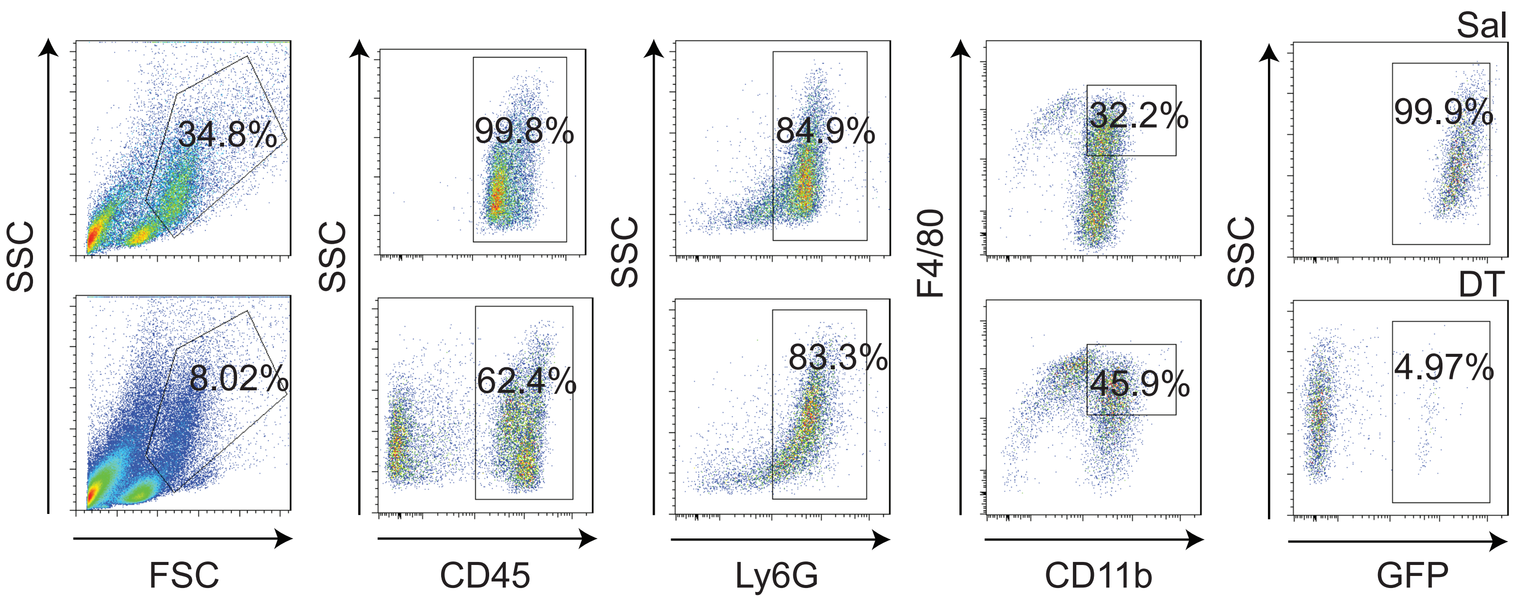

Supplementary Figure6

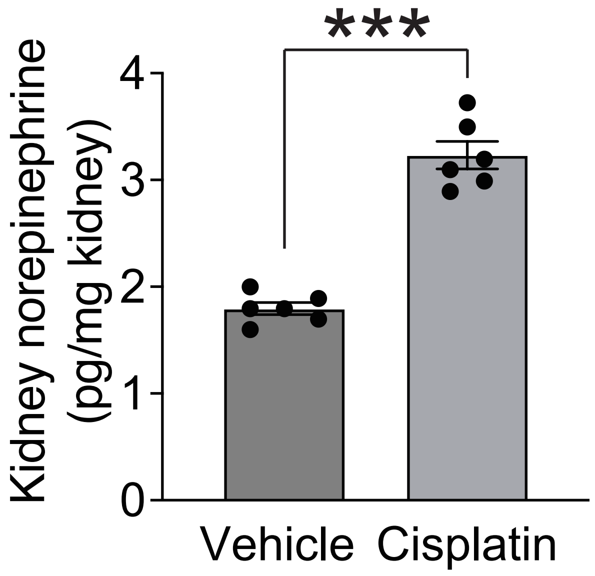

Supplementary Figure7

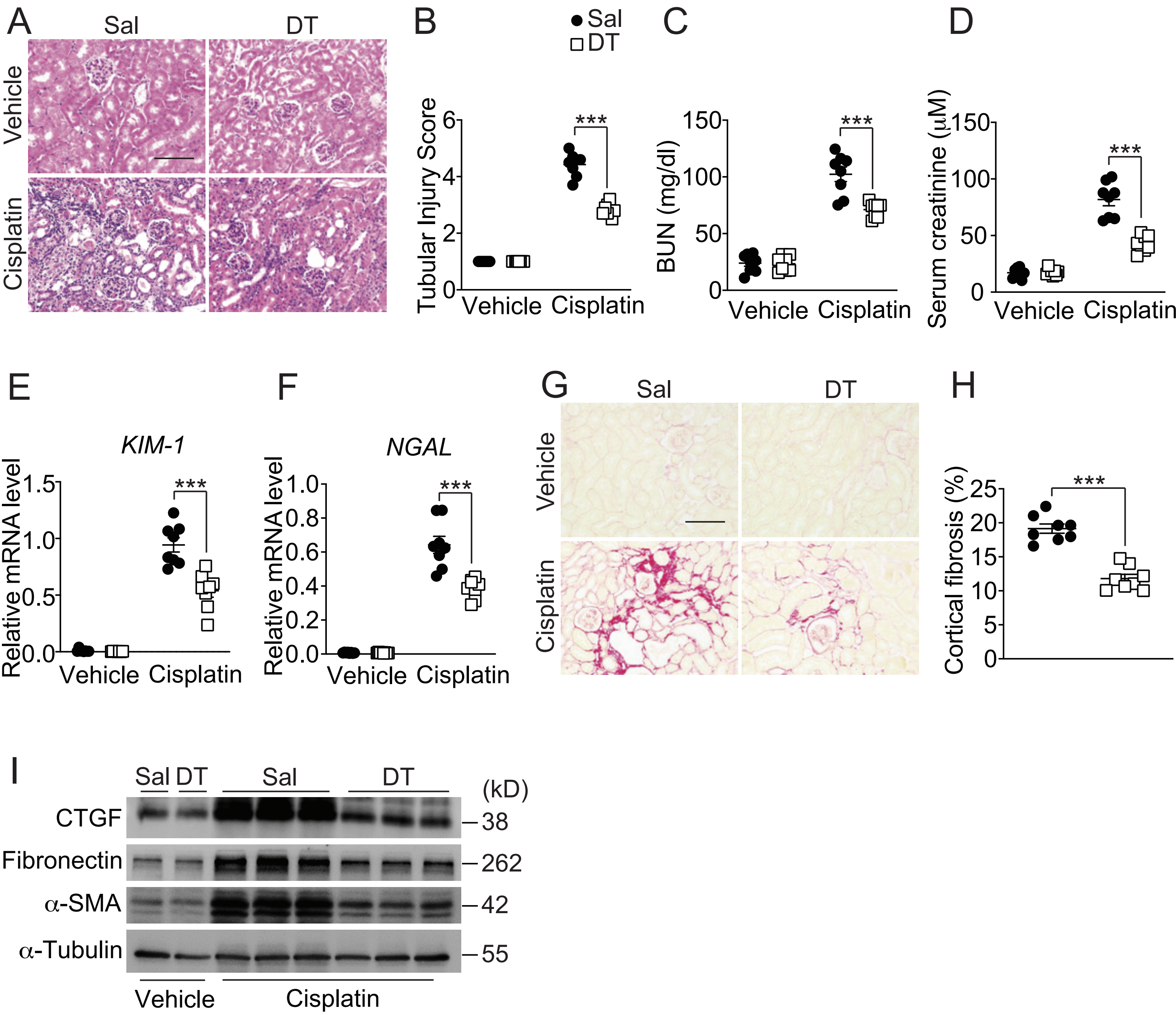

Supplementary Figure8

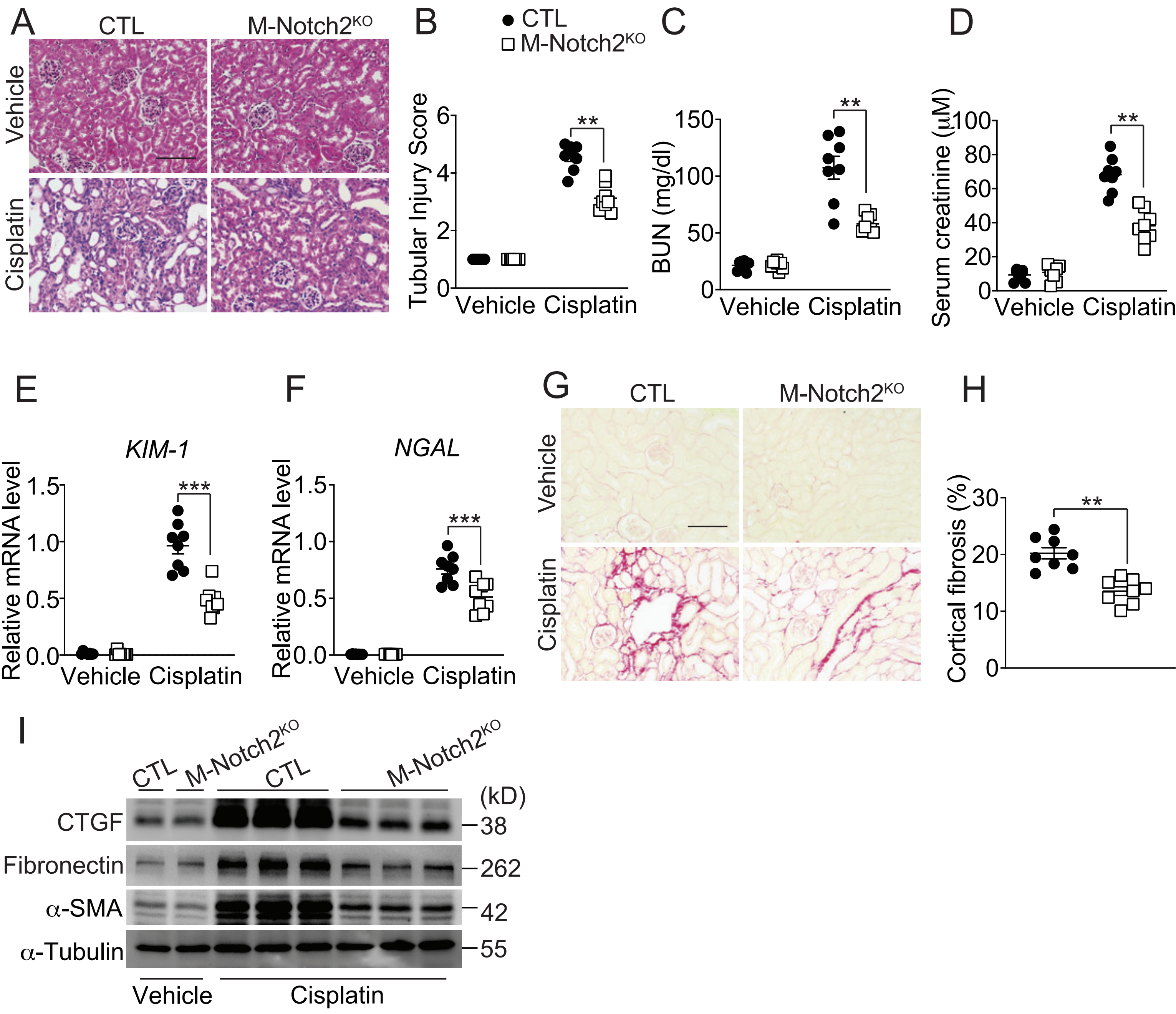

Supplementary Figure9

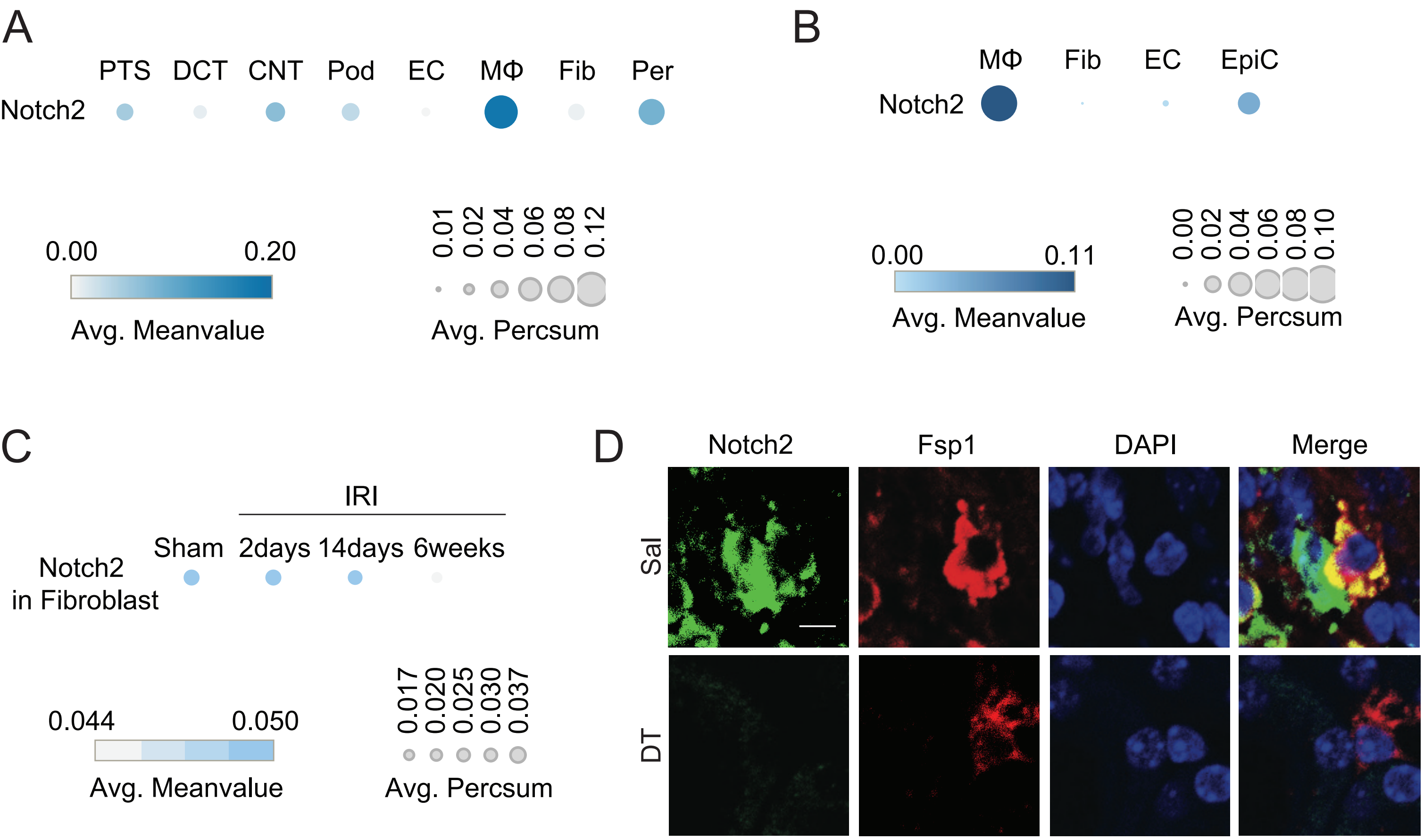

Supplementary　Figure10

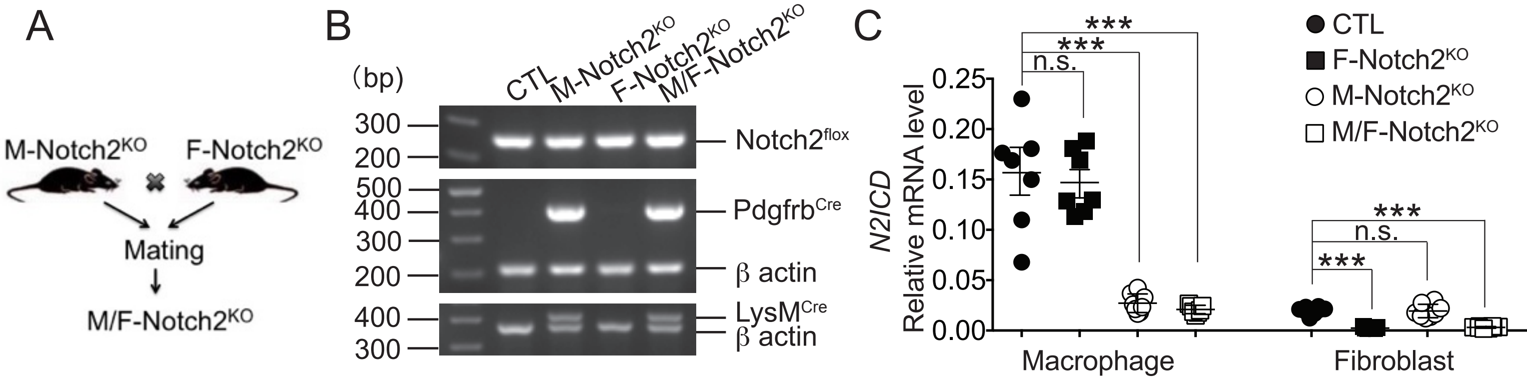

Supplementary Figure11

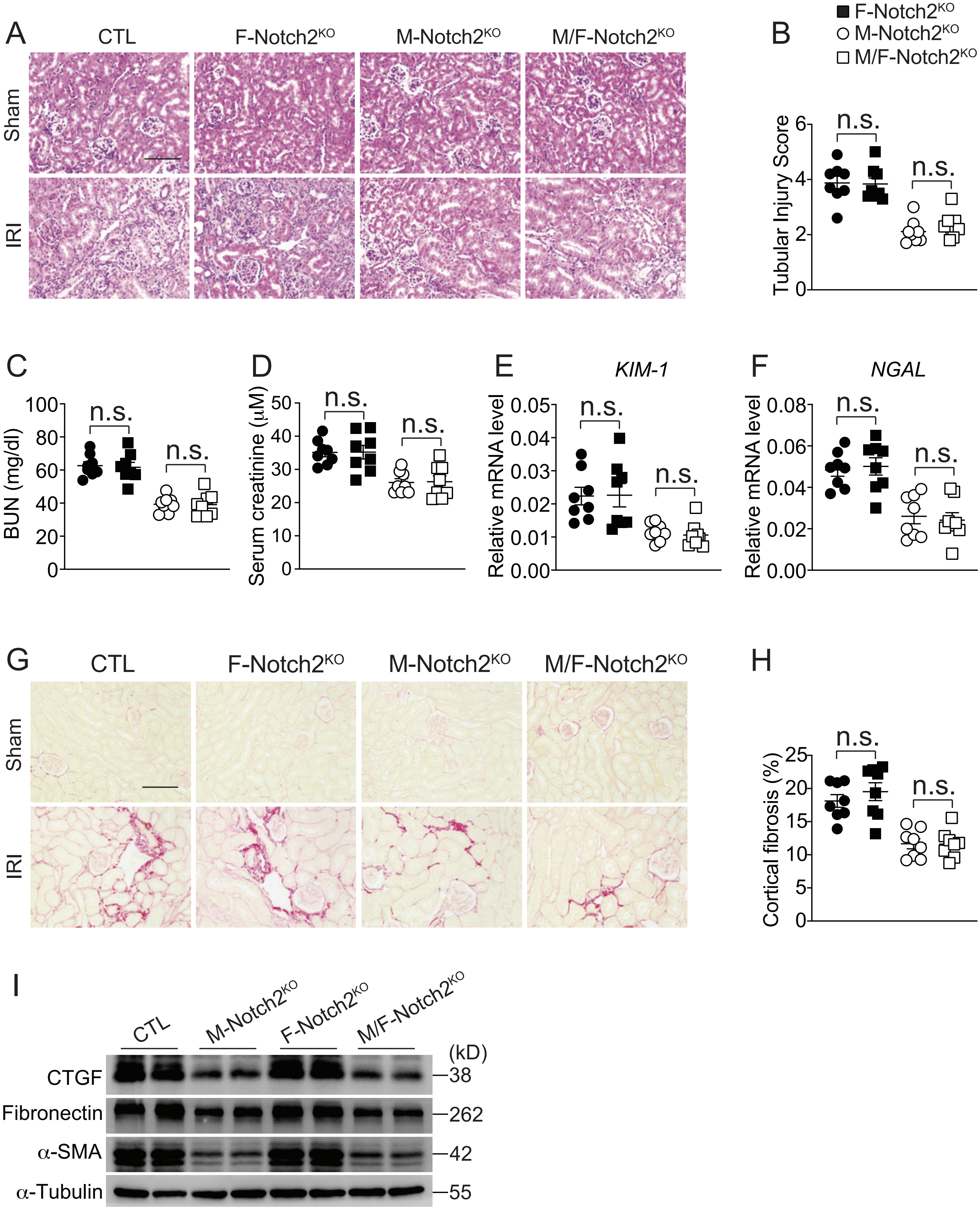

Supplementary Figure12

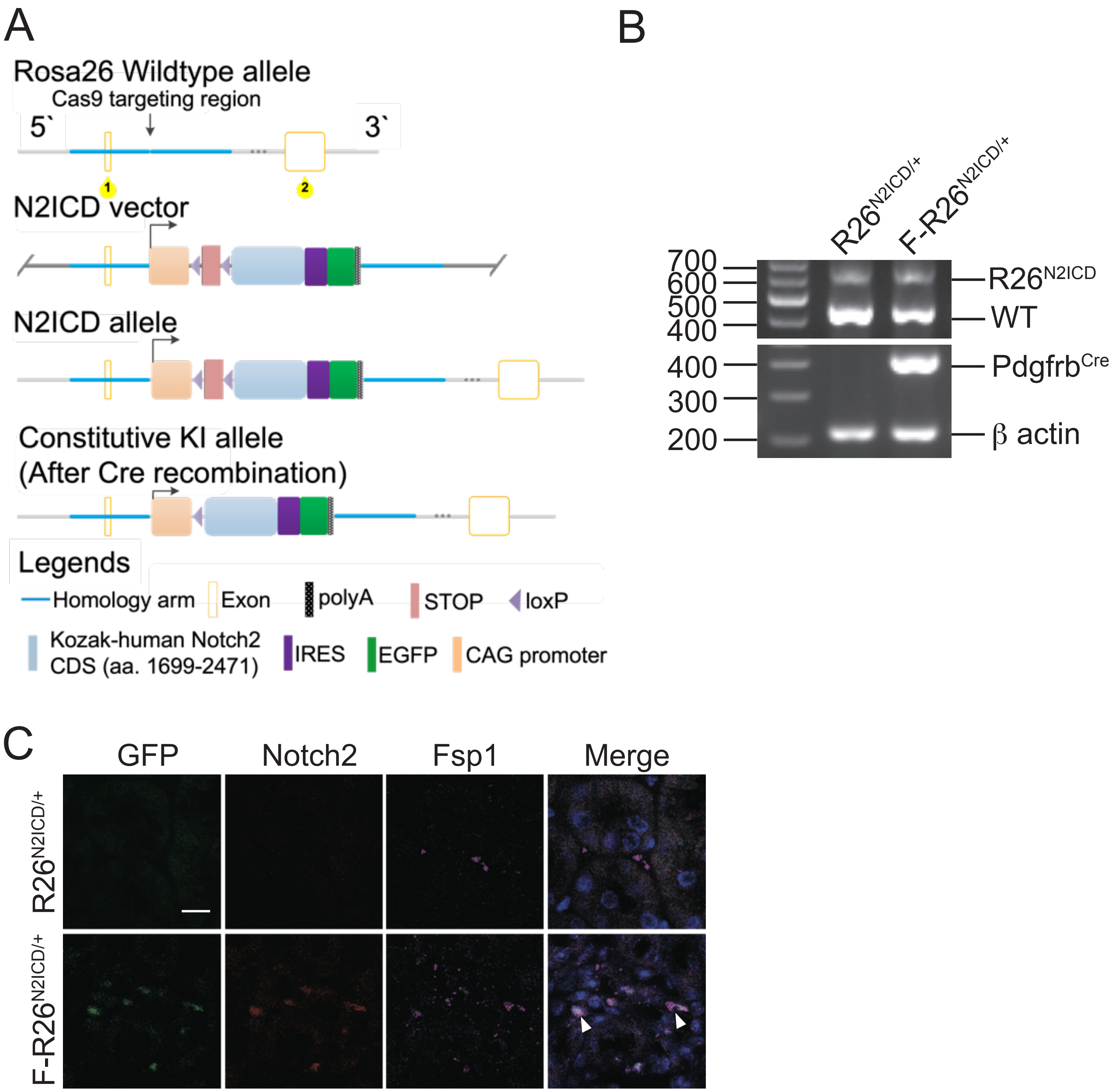

Supplementary Figure13

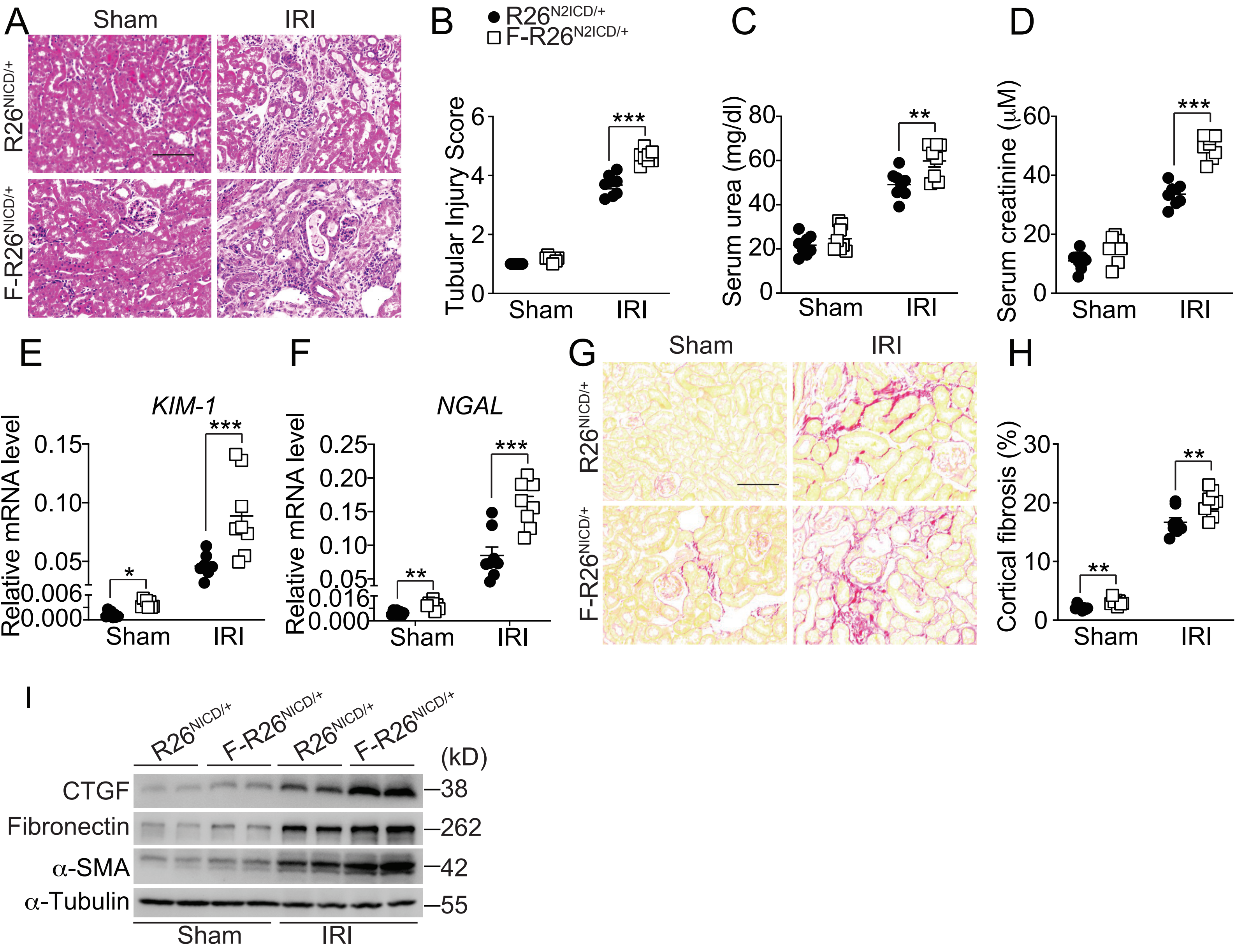

Supplementary Figure14

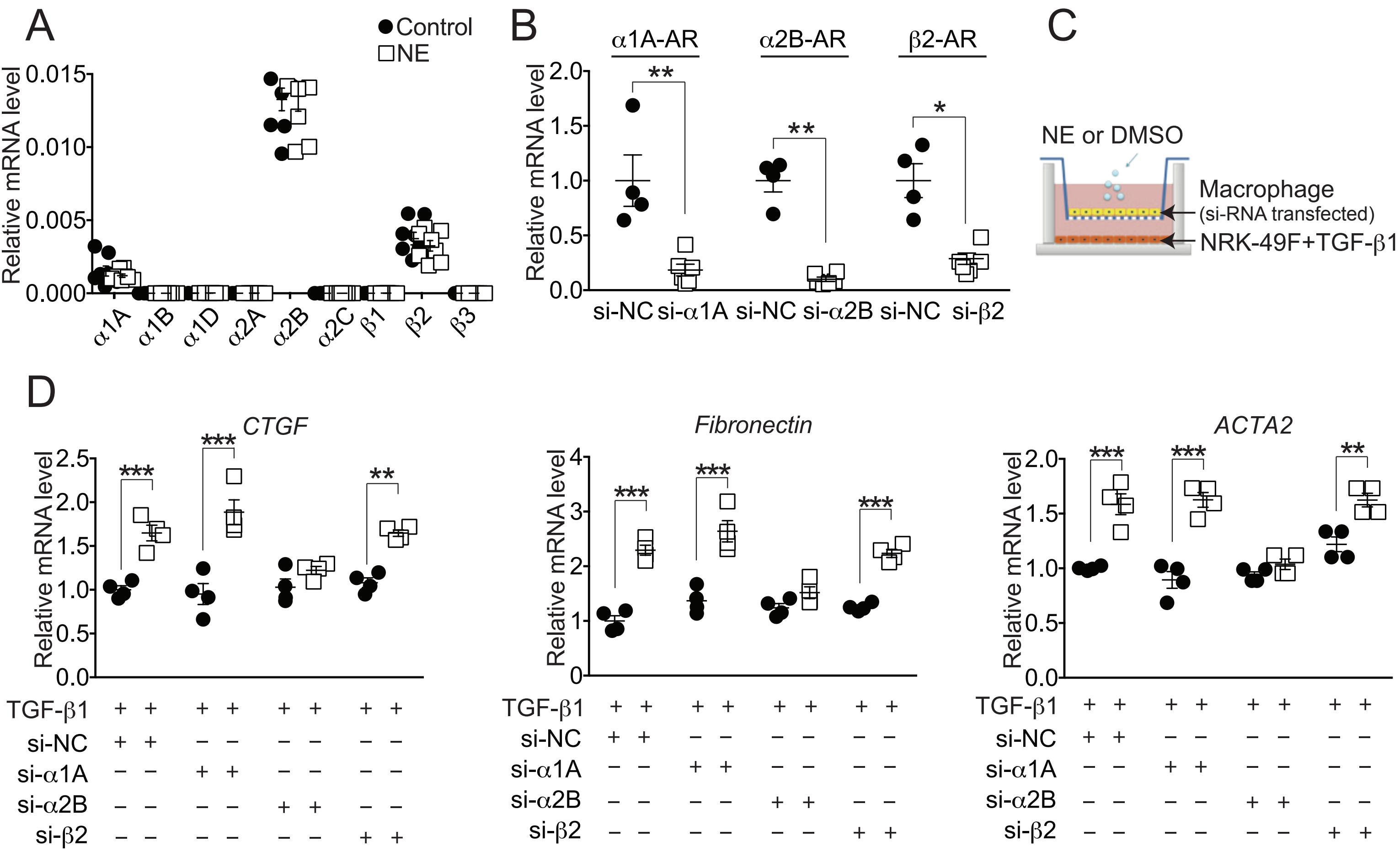

Supplementary Figure15

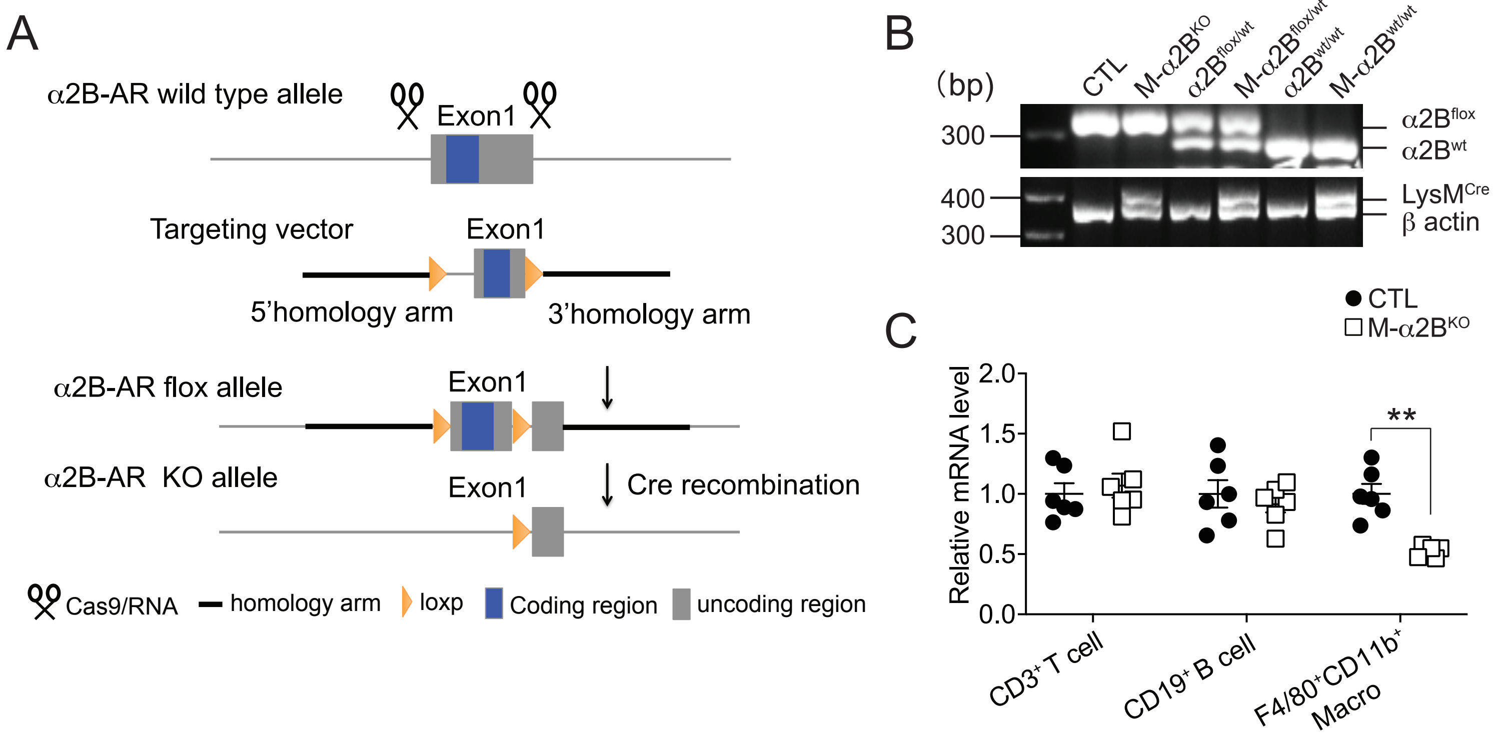

Supplementary Figure16

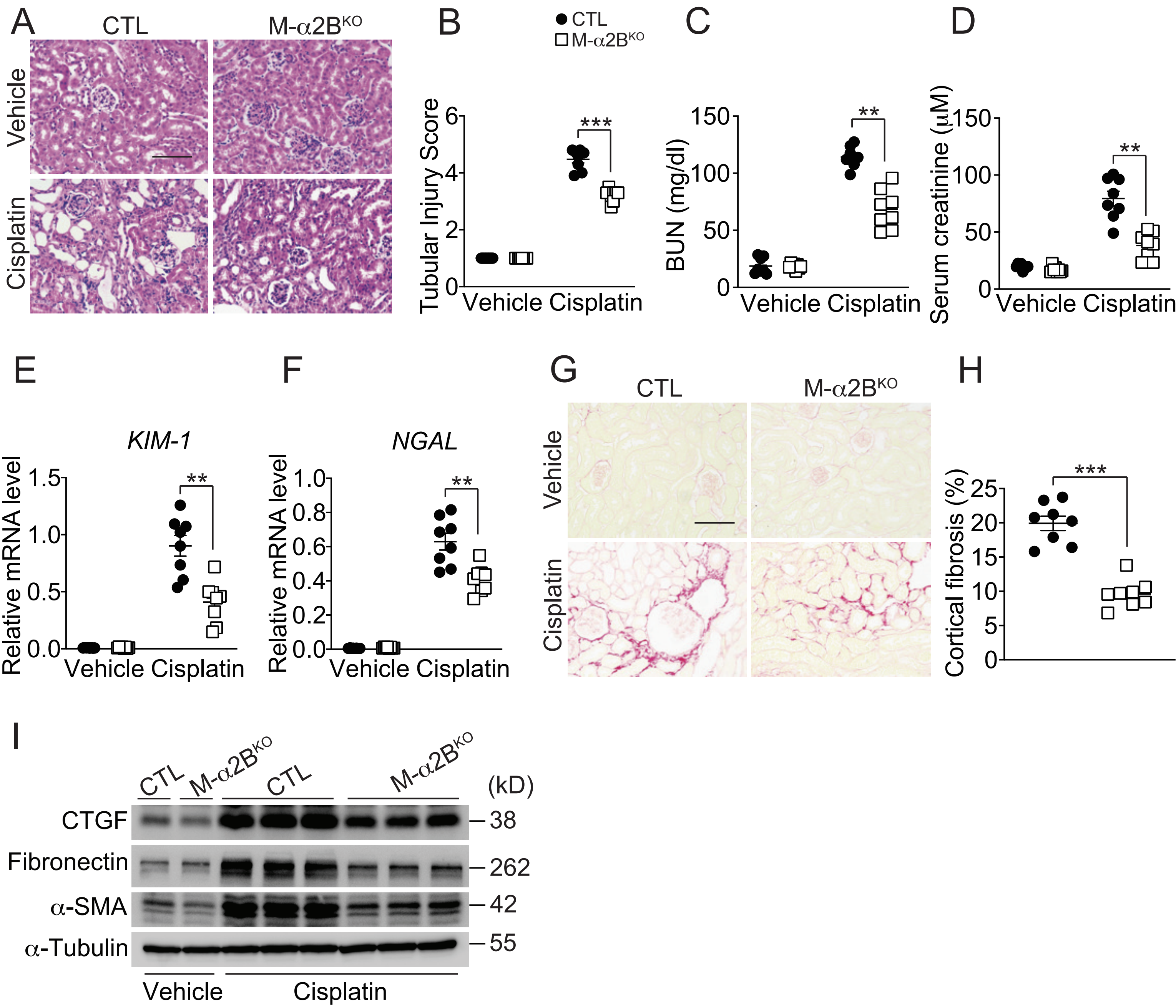

Supplementary Figure17

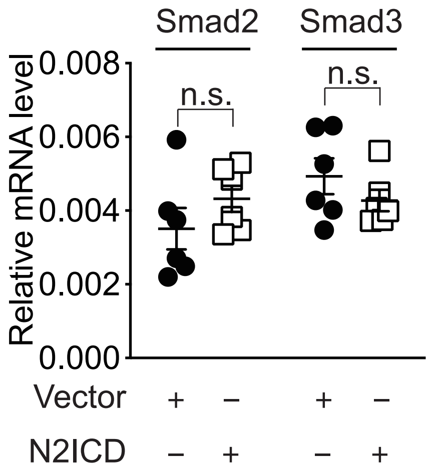

Supplementary Figure18

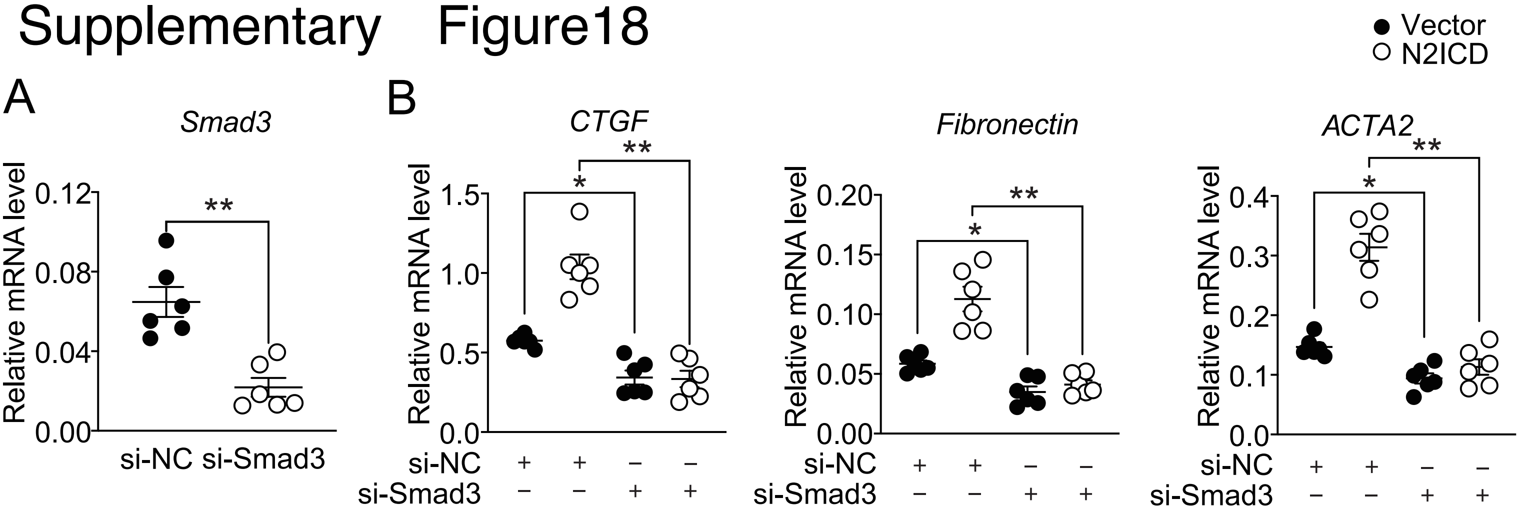

Supplement: Supplementary file 2 — Supporting Information [file ADVS-12-e04607-s001.pdf]
